# Postpartum maternal and infant haematological effects of second-trimester ferric carboxymaltose versus standard-of-care oral iron in Malawi: longitudinal follow-up of a randomised controlled trial

**DOI:** 10.1016/S2214-109X(24)00380-2

**Published:** 2024-11-20

**Authors:** Glory Mzembe, Ernest Moya, Martin N Mwangi, Ricardo Ataide, Rebecca Harding, Justina Kaunda, Truwah Zinenani, Gomezgani Mhango, William Stones, Owen Mtambo, Ayse Y Demir, Hans Verhoef, Sabine Braat, Sant-Rayn Pasricha, Kamija S Phiri

**Affiliations:** a Training and Research Unit of Excellence, Blantyre, Malawi; b School of Public and Global Health, Kamuzu University of Health Sciences, Blantyre, Malawi; c The Micronutrient Forum, Healthy Mothers Healthy Babies Consortium, Washington, DC, USA; d Division of Human Nutrition and Health, Wageningen University, Wageningen, Netherlands; e Population Health and Immunity Division, Walter and Eliza Hall Institute of Medical Research, Parkville, VIC, Australia; f Department of Infectious Diseases, Peter Doherty Institute, University of Melbourne, Parkville, VIC, Australia; g Laboratory for Clinical Chemistry and Haematology, Meander Medical Centre, Amersfoort, Netherlands; h Diagnostic Haematology, The Royal Melbourne Hospital, Parkville, VIC, Australia; i Clinical Haematology, The Peter MacCallum Cancer Centre and The Royal Melbourne Hospital, Parkville, VIC, Australia; j Department of Medical Biology, University of Melbourne, Parkville, VIC, Australia

## Abstract

**Background:**

Anaemia is common in mothers and infants in the first year postpartum, especially in sub-Saharan Africa. We evaluated whether treating anaemia in the second trimester of pregnancy with a single dose of intravenous iron, ferric carboxymaltose, compared with standard-of-care oral iron could alleviate anaemia in postpartum women and their infants.

**Methods:**

REVAMP (ACTRN12618001268235), an open-label, individually randomised, controlled trial done across nine urban and five rural health centres in Malawi, recruited women if they were in the second trimester of singleton pregnancy, had a capillary haemoglobin concentration of less than 10·0 g/dL, and had a negative malaria rapid diagnostic test. Once enrolled, women were randomly assigned (1:1) to receive intravenous ferric carboxymaltose (20 mg/kg up to 1000 mg) or standard of care (60 mg oral elemental iron twice daily for 90 days); all women received preventive malaria treatment. The primary endpoint of REVAMP was anaemia prevalence at 36 weeks of gestation, with follow-up of mothers and infants until 1 month postpartum. In REVAMP-EXTENDED, women from REVAMP who gave consent, and their infants, were followed up at 3, 6, 9, and 12 months postpartum, and venous blood was collected for haemoglobin, ferritin, and C-reactive protein measurement. Maternal postpartum outcomes comprised prevalence of anaemia (venous haemoglobin concentration <11 g/dL up to and including delivery and <12·0 g/dL postpartum) and haemoglobin concentration, as well as iron status (iron deficiency, defined as serum ferritin <15 μg/L, or <30 μg/L if C-reactive protein >5 mg/L, and iron deficiency anaemia [both iron deficiency and anaemia]). Infant outcomes comprised cord ferritin concentration, and haemoglobin and ferritin concentrations at 1, 3, 6, 9, and 12 months of age.

**Findings:**

Between Nov 12, 2018, and March 2, 2021, 862 women were randomly assigned in REVAMP, of whom 793 (393 in the ferric carboxymaltose group [376 liveborn infants] and 400 [379 liveborn infants] in the standard-of-care group) provided consent for REVAMP-EXTENDED. At 12 months postpartum, ferritin concentrations were higher (geometric mean ratio 1·47 [95% CI 1·29–1·66], p<0·0001), and prevalence of iron deficiency was lower (prevalence ratio 0·65 [0·48–0·88], p=0·0050), in mothers receiving ferric carboxymaltose than in those receiving standard of care. Anaemia was less common in women who received ferric carboxymaltose than in those who received standard of care at 1 month (prevalence ratio 0·84 [95% CI 0·71–0·98], p=0·027), 3 months (0·75 [0·62–0·91], p=0·0029), and 6 months (0·78 [0·63–0·96], p=0·018) postpartum but not thereafter. There was no evidence of a difference between groups regarding cord ferritin, infant ferritin, or infant haemoglobin concentrations at any timepoint. Benefits on postpartum anaemia were restricted to mothers with baseline iron deficiency.

**Interpretation:**

Ferric carboxymaltose treatment in the second trimester protected women from postpartum anaemia and iron deficiency but did not affect infant haematological or iron status.

**Funding:**

Bill & Melinda Gates Foundation.

**Translation:**

For the Chichewa translation of the abstract see Supplementary Materials section.

## Introduction

More than 41% of pregnant women worldwide are anaemic,^1^ with the problem particularly affecting women in low-income countries, especially in Asia and sub-Saharan Africa. WHO recommends that pregnant women routinely receive oral iron to prevent iron deficiency anaemia.^2^ However, acceptability and adherence to oral iron during pregnancy are often suboptimal.^3^ An important consequence of antenatal anaemia is postpartum anaemia, which can be exacerbated by blood losses during childbirth.^4^ Postpartum anaemia is a common condition globally, including in sub-Saharan Africa, and compromises wellbeing and might promote postpartum depression.^5^

**Research in context**

**Evidence before this study**

We searched PubMed for “intravenous iron” AND “pregnancy” OR “antenatal”, restricted to clinical trials, from database inception to Feb 6, 2024, without language restrictions. We identified 50 articles, of which 13 reported randomised controlled trials comparing intravenous iron (in various forms) with oral iron to treat anaemia during pregnancy. Most trials followed up women for a relatively short duration (eg, 4–18 weeks). Where conducted, postpartum follow-up was limited to 4 weeks. We also searched systematic reviews investigating intravenous iron compared with oral iron in pregnancy. A network meta-analysis including 30 trials (not only oral *vs* intravenous iron, but also oral *vs* oral iron and intravenous *vs* intravenous iron formulations) identified 4 weeks after infusion as a common follow-up timepoint. One trial reported on the effects of antenatal intravenous iron on cord ferritin concentrations.

**Added value of this study**

To our knowledge, we report the first extended follow-up of the effects of intravenous iron during pregnancy on maternal and infant haematological and iron status in either a low-income or a high-income setting. Our data show that compared with oral iron, intravenous ferric carboxymaltose markedly reduced iron deficiency and iron deficiency anaemia in women up to 12 months postpartum and provided a reduction in anaemia that was sustained for 6 months postpartum; however, there were no effects on the haematological or iron status in infants.

**Implications of all the available evidence**

Intravenous iron acts quicker than oral iron to raise haemoglobin concentration during pregnancy. Our data indicate that high-dose intravenous iron during pregnancy might also provide women with sustained protection from postpartum anaemia and iron deficiency, extending the use case for this therapy across low-income and high-income settings. Trials testing intravenous iron in pregnancy should incorporate extended follow-up to assess postpartum haematological outcomes.

Modern intravenous iron formulations such as ferric carboxymaltose and iron derisomaltose enable high doses (eg, 1000 mg) of iron to be administered in a single, brief (15–30 min) infusion, ensuring delivery of a near total replacement dose of iron and overcoming limitations in adherence to oral iron. In high-income settings, guidelines and expert recommendations increasingly advocate treatment of moderate or severe iron deficiency anaemia in pregnancy with modern intravenous iron formulations.^6^, ^7^ We reasoned that ferric carboxymaltose could present an opportunity to rapidly correct iron deficiency and reduce anaemia prevalence among women living in resource-limited settings in sub-Saharan Africa. The REVAMP trial tested a single dose of up to 1000 mg ferric carboxymaltose compared with oral iron given as standard of care in pregnant women with moderate or severe anaemia in the second trimester of pregnancy living in two districts of Malawi. The trial initially followed up women from recruitment to 1 month postpartum. It did not meet its primary efficacy endpoint, which was predefined as ferric carboxymaltose causing a significant reduction in maternal anaemia prevalence at 36 weeks of gestation compared with standard of care. In infants, ferric carboxymaltose did not increase birthweight.^8^ However, compared with women receiving standard of care, ferric carboxymaltose significantly reduced the prevalence of iron deficiency and iron deficiency anaemia and raised serum ferritin concentrations clinically and statistically at all post-treatment timepoints.

The impact of maternal iron interventions on infant iron and haematological status remains uncertain: evidence largely from observational studies indicates that maternal iron deficiency and anaemia are correlated with reduced infant iron stores, with inconsistent data on associations with infant haemoglobin concentrations and mixed effects from antenatal oral iron interventions.^9^ Maternal iron status might particularly influence infant iron status in the third trimester, when placental iron transfer to the fetus is maximal.^10^ The impact of high-dose intravenous iron given during pregnancy on neonatal and infant iron stores and haematological status has not previously been widely evaluated.

We hypothesised that the increased iron stores over the second and third trimester in mothers through high-dose ferric carboxymaltose could improve postpartum maternal iron stores and alleviate postpartum anaemia compared with standard of care. We also hypothesised that the raised maternal iron stores during pregnancy in the intravenous iron group of the REVAMP trial could drive fetal iron transfer and thus lead to higher infant iron stores and haemoglobin concentration. To test these hypotheses, mothers enrolled in the REVAMP trial who provided appropriate consent (along with their infants) were followed up from 1 month postpartum to 12 months after delivery, for regular assessment of haemoglobin and iron stores.

## Methods

### Study design and participants

REVAMP was an open-label, individually randomised, controlled trial run in Blantyre and Zomba, southern Malawi. The Zomba site served as the base for nine health centres (four urban and five rural), and the Blantyre site served as the base for five health centres (all urban). The primary endpoint of the trial was anaemia prevalence at 36 weeks of gestation, with secondary endpoints in mothers and infants up to and including 1 month postpartum. Full details of the trial protocol, including screening, enrolment, randomisation, follow-up to 1 month postpartum, sample size estimation, and statistical analysis plan, have been published previously.^11^

Briefly, women were eligible if they had moderate or severe anaemia (capillary haemoglobin concentration <10·0 g/dL, but >5·0 g/dL, by HemoCue 301+ [Angelholm, Sweden]), were negative on a rapid diagnostic test (CareStart Malaria HRP2/pLDH [Pf/PAN] COMBO, Access Bio, Somerset, NJ, USA) for *Plasmodium falciparum*, had an ultrasound-confirmed singleton pregnancy at 13–26 weeks of gestation, had no previously diagnosed inherited red cell disorder, and were not clinically considered to urgently require transfusion or have any other serious medical illness requiring urgent hospitalisation. Women underwent screening for eligibility at health centres; eligible women were then centrally randomly assigned (by sealed envelope) in a 1:1 ratio with randomly permuted blocks of size four or six, stratified by site to either the intervention group (a single dose of ferric carboxymaltose [Vifor International, St Gallen, Switzerland] 20 mg/kg, up to 1000 mg, diluted in 250 mL normal saline, intravenously over 15 min) or standard of care (60 mg elemental iron orally as ferrous sulphate, twice daily for 90 days). The trial programme did not provide any participants with either intravenous iron or oral iron supplements postpartum. All participants received intermittent preventive treatment of malaria in pregnancy using sulfadoxine–pyrimethamine at baseline, 28 days after enrolment, and at 36 weeks of gestation unless contraindicated (eg, due to recent malaria therapy [usually with artemether–lumefantrine] or being HIV positive and receiving cotrimoxazole chemoprophylaxis). The trial was approved by ethics committees at the College of Medicine, University of Malawi (Zomba, Malawi), and the Walter and Eliza Hall Institute of Medical Research (Parkville, VIC, Australia); monitored by an independent data and safety monitoring board; and prospectively registered with the Australian New Zealand Clinical Trials Registry, ACTRN12618001268235. All participants gave written informed consent for the extended follow-up, either at baseline or at the 1-month postpartum visit.

Participants were initially followed up at scheduled visits at 28 days post-enrolment, 36 weeks of gestation, during delivery, and 1 month postpartum. For the extended follow-up, REVAMP-EXTENDED, further follow-up visits were conducted at 3, 6, 9, and 12 months postpartum for the women from the original trial who consented to take part.

### Outcomes

Maternal postpartum outcomes comprised prevalence of anaemia (venous haemoglobin concentration <11 g/dL up to and including delivery and <12·0 g/dL postpartum^12^, ^13^, ^14^ measured by a Sysmex [Kobe, Japan] automated analyser) and haemoglobin concentration, as well as iron status (iron deficiency, defined as serum ferritin <15 μg/L, or <30 μg/L if C-reactive protein [CRP] >5 mg/L,^15^, ^16^ and iron deficiency anaemia [both iron deficiency and anaemia]). Infant outcomes comprised cord ferritin concentration, and haemoglobin and ferritin concentrations at 1, 3, 6, 9, and 12 months of age.

### Statistical analysis

The sample size was determined by the number of women who participated in REVAMP, which aimed to detect a 10% reduction in anaemia prevalence at 36 weeks of gestation (60% standard of care *vs* 50% ferric carboxymaltose) in 862 women (80% power, two-sided alpha=5%), with assumed 10% loss to follow-up.^11^ Women and infants with consent to follow-up beyond 1 month postpartum and with available data (ie, contributing at least one non-missing value within allowable visit windows to the model) were analysed. Maternal and infant outcomes were included in the randomly assigned group of the women, and results are presented for all postpartum timepoints. Maternal anaemia, iron deficiency, and iron deficiency anaemia were analysed using a mixed-effects Poisson regression model with a log link, including fixed effects of treatment, study visit, randomised group by postpartum study visit interaction, and site, with a random intercept for the women and robust SEs.^17^ Maternal and infant venous haemoglobin concentration and (log-transformed) serum ferritin concentration were analysed using a linear mixed model by Liang and Zeger,^18^ with a random intercept for participant and an unstructured variance-covariance among the repeated measurements. The independent variables consisted of treatment, randomised group by postpartum study visit interaction, and site. These longitudinal models were fitted to all study visits from baseline to 12 months postpartum for maternal outcomes and from 1 month to 12 months of age for infant outcomes. In addition, this model assumed a common pre-randomisation baseline mean across the two treatment groups for maternal outcomes and included participants with at least one non-missing outcome value. Missing values were not imputed because the model uses a likelihood-based approach to handle missing data that assumes missingness is missing at random among those participants with available data. Neonate (log-transformed) cord ferritin was analysed using a linear regression model with randomised group and site as the independent variables. After exploring imbalances between treatment groups in maternal characteristics at baseline for each maternal and infant outcome separately, all analysis models were adjusted for site (the randomisation stratification factor) only. One subgroup analysis was performed on the maternal baseline characteristic of iron deficiency for postpartum maternal outcomes (anaemia, iron deficiency, iron deficiency anaemia, and haemoglobin and ferritin concentrations) and infant outcomes (haemoglobin and ferritin concentrations), with results presented by subgroup alongside the p value of the subgroup by treatment group interaction test. Results are presented as point estimates, two-sided 95% CIs, and two-sided p values. No multiplicity adjustment is applied because the outcomes in this extended follow-up were not powered for. Analyses were performed using Stata SE (version 18.0).

### Role of the funding source

The funder of the study had no role in study design, data collection, data analysis, data interpretation, or writing of the report.

## Results

Between Nov 12, 2018, and March 2, 2021, 862 women were randomly assigned in REVAMP; the final 12-month postpartum visit was completed on Aug 13, 2022. Because not all participants gave extended consent or were available for consenting at 1 month postpartum, the analysis dataset for all timepoints of the longitudinal follow-up study is a subset of the parent trial (793 mothers [393 in the ferric carboxymaltose group and 400 in the standard-of-care group]; and 755 liveborn infants [376 in the ferric carboxymaltose group and 379 in the standard-of-care group]; appendix 2 p 2). Follow-up and sample collection was achieved for the majority of the women (appendix 2 pp 3–4). Although, as expected, collection of venous blood samples from infants was more difficult, we obtained at least one haemoglobin or serum ferritin sample between 1 month and 12 months of age from about 80% of infants (appendix 2 p 3). Haemoglobin or serum ferritin samples were unavailable for reasons such as refusal of blood sample, dropout, or insufficient sample. However, comparison of baseline maternal characteristics and infant characteristics at delivery indicated no evidence of a difference between children from whom blood was collected compared with the overall sample (appendix 2 pp 4–8).

The difference between the number of participating mothers and the number of liveborn infants is due to factors such as multiple births, pregnancy loss, or loss to follow-up or dropout of mothers who consented for the full duration of the trial at baseline. Table 1 presents baseline characteristics of the analysis dataset for mothers and infants. Characteristics were generally similar between groups. The prevalence of iron deficiency in participating mothers was 42% (328 of 775) at baseline. Participants in the extension study were similar to, and are thus representative of, the parent trial (appendix 2 pp 9–11).

**Table 1:** Baseline characteristics

		**Ferric carboxymaltose group**	**Standard-of-care group**
**Maternal characteristics at enrolment**			
	Mothers	393	400
Site			
	Blantyre	44/393 (11%)	51/400 (13%)
	Zomba	349/393 (89%)	349/400 (87%)
Age, years		22·2 (6·2)	22·5 (6·4)
Primiparous*		212/393 (54%)	219/400 (55%)
Primigravid*		210/393 (53%)	215/400 (54%)
Gestational age, weeks		22·0 (19·7–24·4)	21·9 (18·7–24·1)
Height, cm		155·2 (5·8)	155·3 (7·0)
Weight, kg		55·7 (8·1)	55·3 (8·6)
BMI, kg/m^2^		23·1 (2·9)	22·9 (3·2)
Religion*			
	None	1/392 (<1%)	1/398 (<1%)
	Christian	265/392 (68%)	298/398 (75%)
	Muslim	123/392 (31%)	94/398 (24%)
	Other	3/392 (1%)	5/398 (1%)
Education*			
	None	1/381 (<1%)	1/382 (<1%)
	Lower primary	84/381 (22%)	81/382 (21%)
	Upper primary	162/381 (43%)	160/382 (42%)
	Lower secondary	48/381 (13%)	57/382 (15%)
	Upper secondary	77/381 (20%)	74/382 (19%)
	Tertiary	9/381 (2%)	9/382 (2%)
Marital status*			
	Single	60/392 (15%)	66/398 (17%)
	Married	328/392 (84%)	321/398 (81%)
	Widowed	0/392 (0%)	3/398 (1%)
	Divorced or separated	4/392 (1%)	6/398 (2%)
	Other	0/392 (0%)	2/398 (1%)
Income source*			
	None	22/392 (6%)	29/398 (7%)
	Subsistence farming	78/392 (20%)	71/398 (18%)
	Large-scale farming	1/392 (<1%)	1/398 (<1%)
	Employed	68/392 (17%)	58/398 (15%)
	Casual work for wages	128/392 (33%)	121/398 (30%)
	Business	90/392 (23%)	112/398 (28%)
	Other	5/392 (1%)	6/398 (2%)
Positive malaria RDT (for *Plasmodium falciparum* parasitaemia)†		6/383 (2%)	6/394 (2%)
HIV positive*		65/390 (17%)	68/396 (17%)
Capillary haemoglobin <10 g/dL		392/392 (100%)	399/399 (100%)
Venous haemoglobin‡, g/dL		8·78 (1·23)	8·85 (1·23)
Anaemia based on venous haemoglobin			
	No (≥11 g/dL)	17/389 (4%)	18/400 (5%)
	Mild (10 g/dL to <11 g/dL)	42/389 (11%)	52/400 (13%)
	Moderate (7 g/dL to <10 g/dL)	302/389 (78%)	303/400 (76%)
	Severe (<7 g/dL)	28/389 (7%)	27/400 (7%)
Serum ferritin§, μg/L		26·6 (10·0–78·7)	28·8 (10·7–64·9)
CRP§, mg/L		5·25 (2·70–11·60)	5·10 (2·90–10·60)
Iron deficiency¶		163/382 (43%)	165/393 (42%)
Iron deficiency anaemia‖		153/378 (40%)	159/393 (40%)
Inflammation**		197/382 (52%)	198/393 (50%)
Anaemia and inflammation††		188/378 (50%)	188/393 (48%)
**Neonate characteristics at delivery**			
Neonates		376	379
Birthweight‡‡, g		2891·6 (500·9)	2894·2 (516·2)
Birth length§§, cm		47·6 (3·5)	47·5 (3·7)
Sex			
	Female	178/371 (48%)	178/365 (49%)
	Male	193/371 (52%)	187/365 (51%)
Gestation duration¶¶, weeks		39·9 (38·6–40·8)	39·7 (38·6–40·8)

Data are n, n/N (%), mean (SD), or median (IQR). Percentages might not sum to 100 as a result of rounding. Two mothers in each group had multiple births, and four twins were born in each group. CRP=C-reactive protein. RDT=rapid diagnostic test.

* Self-reported.

† A positive malaria RDT was based on confirmatory testing by laboratory personnel on venous blood collected at enrolment.

‡ Data are missing for four participants in the ferric carboxymaltose group and no participants in the standard-of-care group.

§ Data are missing for 11 participants in the ferric carboxymaltose group and seven participants in the standard-of-care group.

¶ Iron deficiency is defined as serum ferritin less than 15 μg/L, or less than 30 μg/L if CRP is higher than 5 mg/L.

‖ Iron deficiency anaemia is defined as venous haemoglobin less than 11 g/dL and serum ferritin less than 15 μg/L, or serum ferritin concentration less than 30 μg/L if CRP is higher than 5 mg/L.

** Inflammation is defined as CRP higher than 5 mg/L.

†† Anaemia and inflammation is defined as venous haemoglobin less than 11·0 g/dL up to and including delivery and CRP higher than 5 mg/L.

‡‡ Data are missing for six participants in the ferric carboxymaltose group and 17 participants in the standard-of-care group.

§§ Data are missing for 14 participants in the ferric carboxymaltose group and 21 participants in the standard-of-care group.

¶¶ Data are missing for four participants in the ferric carboxymaltose group and 11 participants in the standard-of-care group.

At 1, 3, and 6 months postpartum, compared with women receiving standard of care, anaemia was less common in women who received ferric carboxymaltose (prevalence ratio 0·84 [95% CI 0·71–0·98], p=0·027, at 1 month; 0·75 [0·62–0·91], p=0·0029, at 3 months; and 0·78 [0·63–0·96], p=0·018, at 6 months; table 2). Moderate or severe anaemia (haemoglobin concentration <10 g/dL) was less common among women who received ferric carboxymaltose than among those who received standard of care at 3 months postpartum (prevalence ratio 0·67 [95% CI 0·46–1·00, p=0·049) and 6 months postpartum (0·65 [0·43–0·97], p=0·035). Maternal haemoglobin concentrations were higher at 1 month and 3 months postpartum in the ferric carboxymaltose group than in the standard-of-care group (mean difference 0·27 g/dL [95% CI 0·07–0·48], p=0·0071, at 1 month *vs* 0·26 g/dL [0·07–0·45], p=0·0077, at 3 months). At all timepoints from 1 month to 12 months postpartum, the prevalence of iron deficiency and iron deficiency anaemia was lower, and mean ferritin concentration was higher, in the ferric carboxymaltose group than in the standard-of-care group.

**Table 2:** Maternal postpartum outcomes

	**Ferric carboxymaltose group (n=393)**	**Standard-of-care group (n=400)**	Prevalence ratio, mean difference, or geometric mean ratio (95% CI*)	p value*
**Anaemia**†‡				
1 month postpartum	140/306 (46%)	166/307 (54%)	Prevalence ratio 0·84 (0·71 to 0·98)	0·027
3 months postpartum	101/250 (40%)	128/243 (53%)	Prevalence ratio 0·75 (0·62 to 0·91)	0·0029
6 months postpartum	97/290 (33%)	119/278 (43%)	Prevalence ratio 0·78 (0·63 to 0·96)	0·018
9 months postpartum	111/275 (40%)	111/262 (42%)	Prevalence ratio 0·95 (0·78 to 1·16)	0·60
12 months postpartum	118/293 (40%)	102/270 (38%)	Prevalence ratio 1·06 (0·86 to 1·30)	0·58
**Moderate or severe anaemia**†§				
1 month postpartum	63/306 (21%)	67/307 (22%)	Prevalence ratio 0·90 (0·67 to 1·23)	0·52
3 months postpartum	34/250 (14%)	48/243 (20%)	Prevalence ratio 0·67 (0·46 to 1·00)	0·049
6 months postpartum	33/290 (11%)	50/278 (18%)	Prevalence ratio 0·65 (0·43 to 0·97)	0·035
9 months postpartum	42/275 (15%)	38/262 (15%)	Prevalence ratio 1·05 (0·71 to 1·57)	0·79
12 months postpartum	42/293 (14%)	37/270 (14%)	Prevalence ratio 1·05 (0·70 to 1·58)	0·81
**Iron deficiency**†¶				
1 month postpartum	36/286 (13%)	86/298 (29%)	Prevalence ratio 0·39 (0·27 to 0·55)	<0·0001
3 months postpartum	30/240 (12%)	63/250 (25%)	Prevalence ratio 0·42 (0·29 to 0·61)	<0·0001
6 months postpartum	40/267 (15%)	73/252 (29%)	Prevalence ratio 0·45 (0·32 to 0·63)	<0·0001
9 months postpartum	46/260 (18%)	77/260 (30%)	Prevalence ratio 0·51 (0·37 to 0·70)	<0·0001
12 months postpartum	57/257 (22%)	73/255 (29%)	Prevalence ratio 0·65 (0·48 to 0·88)	0·0050
**Iron deficiency anaemia**†‖				
1 month postpartum	25/273 (9%)	59/271 (22%)	Prevalence ratio 0·36 (0·23 to 0·56)	<0·0001
3 months postpartum	11/228 (5%)	44/226 (19%)	Prevalence ratio 0·21 (0·11 to 0·38)	<0·0001
6 months postpartum	24/258 (9%)	44/239 (18%)	Prevalence ratio 0·43 (0·27 to 0·68)	<0·0001
9 months postpartum	23/252 (9%)	46/239 (19%)	Prevalence ratio 0·39 (0·25 to 0·62)	<0·0001
12 months postpartum	34/253 (13%)	39/235 (17%)	Prevalence ratio 0·64 (0·41 to 0·98)	0·039
Venous haemoglobin concentration**				
1 month postpartum	11·95 g/dL (1·37); n=306	11·76 g/dL (1·30); n=307	Mean difference 0·27 g/dL (0·07 to 0·48)	0·0071
3 months postpartum	12·11 g/dL (1·15); n=250	11·86 g/dL (1·27); n=243	Mean difference 0·26 g/dL (0·07 to 0·45)	0·0077
6 months postpartum	12·25 g/dL (1·14); n=290	12·08 g/dL (1·41); n=278	Mean difference 0·16 g/dL (−0·04 to 0·35)	0·11
9 months postpartum	12·08 g/dL (1·18); n=275	12·15 g/dL (1·26); n=262	Mean difference −0·07 g/dL (−0·26 to 0·12)	0·48
12 months postpartum	12·12 g/dL (1·31); n=293	12·11 g/dL (1·34); n=270	Mean difference 0·02 g/dL (−0·18 to 0·23)	0·83
**Serum ferritin concentration**††				
1 month postpartum	68·5 μg/L (32·0 to 126·1); n=286	31·3 μg/L (14·8 to 63·2); n=298	Geometric mean ratio 1·92 (1·68 to 2·20)	<0·0001
3 months postpartum	55·4 μg/L (27·3 to 104·1); n=240	26·4 μg/L (16·7 to 46·3); n=250	Geometric mean ratio 1·89 (1·65 to 2·16)	<0·0001
6 months postpartum	43·3 μg/L (23·2 to 85·8); n=267	27·7 μg/L (14·2–45·0); n=252	Geometric mean ratio 1·73 (1·54 to 1·94)	<0·0001
9 months postpartum	40·4 μg/L (20·9 to 85·9); n=260	24·3 μg/L (14·2 to 39·4); n=260	Geometric mean ratio 1·66 (1·48 to 1·87)	<0·0001
12 months postpartum	38·0 μg/L (16·4 to 78·1); n=257	26·3 μg/L (14·9 to 42·8); n=255	Geometric mean ratio 1·47 (1·29 to 1·66)	<0·0001

Data are n/N (%), mean (SD), or median (IQR) unless otherwise specified. The analysis set is restricted to those randomly assigned women who consented for the extended follow-up of the trial to 12 months postpartum and with at least one outcome from enrolment to 12 months postpartum.

* The p values and 95% CIs presented have not been adjusted for multiple comparisons.

† A prevalence ratio of ferric carboxymaltose versus standard of care is shown for anaemia, moderate or severe anaemia, iron deficiency, and iron deficiency anaemia at 1, 3, 6, 9, and 12 months postpartum following analyses using a Poisson model with random intercept for the women and robust SEs; the p value of the interaction between randomised group and postpartum study visits was 0·027 for anaemia, 0·16 for moderate or severe anaemia, 0·0002 for iron deficiency, and 0·0001 for iron deficiency anaemia.

‡ Anaemia is defined as venous haemoglobin less than 11·0 g/dL up to and including delivery and venous haemoglobin less than 12·0 g/dL postpartum.

§ Moderate or severe anaemia is defined as venous haemoglobin less than 10 g/dL up to and including delivery and venous haemoglobin less than 11·0 g/dL postpartum.

¶ Iron deficiency is defined as serum ferritin less than 15 μg/L, or less than 30 μg/L if C-reactive protein is higher than 5 mg/L.

‖ Iron deficiency anaemia is defined as venous haemoglobin less than 11 g/dL up to and including delivery and serum ferritin less than 15 μg/L, or serum ferritin less than 30 μg/L if C-reactive protein is higher than 5 mg/L.

** An absolute mean difference of ferric carboxymaltose versus standard of care of the estimated change from baseline to 1, 3, 6, 9, and 12 months postpartum is shown for continuous haemoglobin following analyses using a longitudinal data analysis model (including all timepoints from baseline to 12 months postpartum). The p value of the interaction between randomised group and postpartum study visits was 0·0043.

†† A geometric mean ratio of ferric carboxymaltose versus standard of care of the estimated change from baseline to 1, 3, 6, 9, and 12 months postpartum is shown for continuous (log-transformed) ferritin following analyses using a longitudinal data analysis model (including all timepoints from baseline to 12 months postpartum). The p value of the interaction between randomised group and postpartum study visits was less than 0·0001.

There was no evidence of a difference in haemoglobin concentration between infants in the ferric carboxymaltose group compared with those in the standard-of-care group (table 3). Likewise, there was no evidence of a difference between groups in ferritin concentrations in cord blood or over the first 12 months of life. There was no evidence of differences between breastfeeding practices between groups (appendix 2 p 12).

**Table 3:** Infant outcomes

	**Ferric carboxymaltose group (n=315)**	**Standard-of-care group (n=315)**	Mean difference or geometric mean ratio (95% CI*)	p value*
**Venous haemoglobin concentration**†				
1 month	11·94 g/dL (1·80); n=207	11·90 g/dL (1·85); n=202	Mean difference 0·10 g/dL (−0·24 to 0·45)	0·56
3 months	10·30 g/dL (1·26); n=170	10·50 g/dL (1·09); n=151	Mean difference −0·17 g/dL (−0·41 to 0·07)	0·17
6 months	10·08 g/dL (1·28); n=180	10·20 g/dL (1·14); n=170	Mean difference −0·13 g/dL (−0·36 to 0·11)	0·29
9 months	9·89 g/dL (1·30); n=178	9·85 g/dL (1·10); n=156	Mean difference 0·03 g/dL (−0·21 to 0·27)	0·82
12 months	9·97 g/dL (1·23); n=199	10·03 g/dL (1·20); n=183	Mean difference −0·02 g/dL (−0·26 to 0·21)	0·84
**Serum ferritin concentration**‡				
1 month	244·6 μg/L (172·9 to 382·9); n=143	223·9 μg/L (157·9 to 347·9); n=143	Geometric mean ratio 1·02 (0·87 to 1·21)	0·80
3 months	111·7 μg/L (64·2 to 171·6); n=102	104·9 μg/L (60·4 to 168·2); n=84	Geometric mean ratio 1·06 (0·87 to 1·29)	0·57
6 months	27·3 μg/L (13·9 to 52·7); n=106	29·7 μg/L (16·6 to 46·6); n=108	Geometric mean ratio 0·96 (0·78 to 1·17)	0·68
9 months	17·7 μg/L (8·2 to 28·8); n=94	16·8 μg/L (9·9 to 28·5); n=71	Geometric mean ratio 1·00 (0·80 to 1·26)	0·97
12 months	14·8 μg/L (8·4 to 24·2); n=95	15·2 μg/L (8·9 to 26·2); n=85	Geometric mean ratio 0·85 (0·68 to 1·06)	0·16
**Cord ferritin concentration**§				
At birth	174·2 μg/L (110·9 to 255·1); n=280	156·0 μg/L (93·9 to 237·0); n=289	Geometric mean ratio 1·10 (0·95 to 1·27)	0·19

Data are n/N (%), mean (SD), or median (IQR) unless otherwise specified. The analysis set is restricted to liveborn children whose mothers were randomly assigned and consented for the extended follow-up of the trial to 12 months postpartum and with at least one outcome from 1 month to 12 months of age.

* The p values and 95% CIs presented have not been adjusted for multiple comparisons.

† An absolute mean difference of ferric carboxymaltose versus standard of care for continuous haemoglobin at 1, 3, 6, 9, and 12 months of age is shown following analyses using a longitudinal data analysis model. The p value of the interaction between randomised group and age of the infant is 0·48.

‡ A geometric mean ratio of ferric carboxymaltose versus standard of care for continuous (log-transformed) ferritin at 1, 3, 6, 9, and 12 months of age is shown following analyses using a longitudinal data analysis model. The p value of the interaction between randomised group and age of the infant is 0·56.

§ A geometric mean ratio for (log-transformed) cord ferritin at delivery between ferric carboxymaltose versus standard of care is shown following fitting a linear regression model.

**Figure 1:**
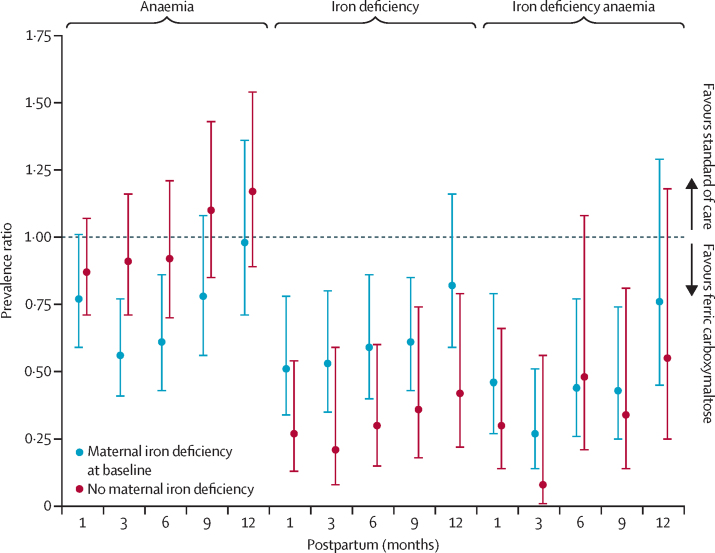
Maternal outcomes by maternal iron deficiency status at baseline Anaemia is defined as venous haemoglobin less than 11·0 g/dL up to and including delivery and venous haemoglobin less than 12·0 g/dL postpartum. Iron deficiency is defined as serum ferritin less than 15 μg/L, or less than 30 μg/L if C-reactive protein is higher than 5 mg/L. Iron deficiency anaemia indicates venous haemoglobin less than 11g/dL up to and including delivery and serum ferritin less than 15 μg/L, or serum ferritin less than 30 μg/L if C-reactive protein is higher than 5 mg/L. A prevalence ratio (error bars indicate 95% CIs) of ferric carboxymaltose versus standard of care is plotted for maternal anaemia, iron deficiency, and iron deficiency anaemia at 1, 3, 6, 9, and 12 months postpartum.

**Figure 2:**
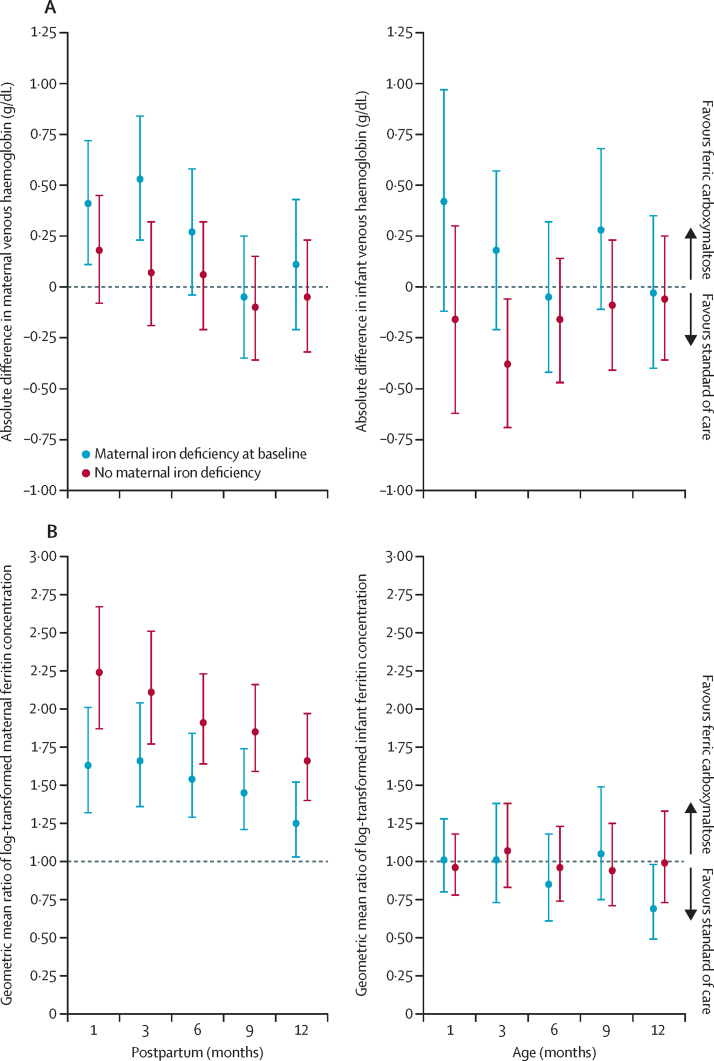
Maternal and infant outcomes by maternal iron deficiency status at baseline (A) Maternal and infant venous haemoglobin. An absolute mean difference (error bars indicate 95% CIs) of ferric carboxymaltose versus standard of care of the estimated change from baseline to 1, 3, 6, 9, and 12 months postpartum is plotted for maternal and infant haemoglobin following analyses using a longitudinal data analysis model. (B) Maternal and infant serum ferritin. A geometric mean ratio (error bars indicate 95% CIs) is displayed for maternal and infant log-transformed ferritin concentration. Anaemia is defined as venous haemoglobin less than 11·0 g/dL up to and including delivery and venous haemoglobin less than 12·0 g/dL postpartum. Iron deficiency is defined as serum ferritin less than 15 μg/L, or less than 30 μg/L if C-reactive protein is higher than 5 mg/L. Iron deficiency anaemia is defined as venous haemoglobin less than 11g/dL up to and including delivery and serum ferritin less than 15 μg/L, or serum ferritin less than 30 μg/L if C-reactive protein is higher than 5 mg/L.

Finally, in a subgroup analysis, we evaluated whether maternal iron deficiency at baseline influenced the effect of ferric carboxymaltose on postpartum maternal and infant haematological and iron outcomes (appendix 2 pp 13–16). In women with baseline iron deficiency, ferric carboxymaltose (compared with standard of care) resulted in larger reductions in anaemia at 3 months and 6 months postpartum; conversely, women without baseline iron deficiency did not show a difference in anaemia prevalence with ferric carboxymaltose (figure 1). Similarly, improvement in haemoglobin concentrations up to 6 months postpartum was higher in women with baseline iron deficiency receiving ferric carboxymaltose than in those receiving standard of care, whereas a benefit was not evident in women without baseline iron deficiency (figure 2A). However, there was no definite evidence of a difference in haemoglobin concentration between infants in the ferric carboxymaltose and those in the standard-of-care group, regardless of the mother's baseline iron status (figure 2A). Similarly, there was no evidence of an interaction between iron deficiency during the second trimester of pregnancy, intervention group, and infant ferritin concentration up to 12 months postpartum (figure 1; figure 2B).

## Discussion

Treatment of women in Malawi with moderate or severe anaemia in the second trimester of pregnancy with intravenous ferric carboxymaltose (compared with oral iron) reduced the prevalence of anaemia and increased haemoglobin concentration from 1 month to 6 months postpartum and reduced iron deficiency and iron deficiency anaemia up to 12 months postpartum. Notably, these maternal gains due to ferric carboxymaltose were not reflected in significantly higher infant haemoglobin concentrations, or cord or infant ferritin concentrations, in this setting. Benefits from intravenous iron were greater in women with baseline iron deficiency than in those without.

Postpartum anaemia is a common and important condition with widespread potential adverse effects that can impair maternal and, subsequently, infant health. Prevention of postpartum anaemia is crucial because it is associated with important outcomes, including postpartum depression,^19^ reduced maternal wellbeing, and impaired mother–infant bonding.^5^ Intravenous iron (given postpartum) has been successfully tested as a treatment for postpartum anaemia.^20^ Postpartum anaemia is driven by underlying antenatal iron deficiency and iron deficiency anaemia, aggravated by blood loss during and after childbirth, and perpetuated by postpartum iron deficiency.^4^, ^21^ Our data suggest that antenatal treatment with high-dose intravenous iron might break this cycle by enhancing postpartum iron stores and reducing the risk of anaemia. The effect on anaemia at 6 months postpartum represents a sustained effect, occurring as late as 12 months after the intervention.

The transfer of iron between mother and child is thought to occur mainly in the third trimester.^10^ Although women receiving ferric carboxymaltose in the second trimester had a higher ferritin concentration across the third trimester of pregnancy than women in the standard-of-care group, we could not find evidence of increased iron endowment among children born to these mothers. Several human studies have indicated correlations between maternal iron concentrations and cord or infant iron concentrations when using ferritin as a surrogate, but the causality of this relationship has been uncertain.^9^, ^22^ A trial in the USA showed that, compared with oral iron, antenatal intravenous iron (given as ferumoxytol) raised cord ferritin concentrations.^23^, ^24^ Likewise, in a randomised trial in Kenya, cord ferritin concentrations were raised at birth in infants born to women who received oral iron during pregnancy compared with placebo.^25^ Potential explanations for discrepant findings with our study include a high prevalence of inflammation in participants (eg, 36% had increased CRP at the time of delivery), potentially impairing iron transfer, which aligns with preclinical observations that placental maternal–fetal iron transfer might be restricted by inflammation.^26^, ^27^ Intravenous iron might potentiate inflammation-induced increases in maternal hepcidin, impairing placental iron transfer. Alternatively, the total amount of iron transfer to the fetus might already have been saturated, restricting additional transfer in the intervention group. Exclusive breastfeeding can affect infant iron stores. In our trial, women in the ferric carboxymaltose group and those in the standard-of-care group did not report any differences in their breastfeeding practices; therefore, this is not likely to have contributed to the infant outcomes. Ultimately, the evidence suggests that, although high-dose iron supplementation in the second trimester increases maternal iron stores during pregnancy, there is no evidence of a corresponding significant increase in iron transfer to the fetus. This finding has important implications for clinical practice and should be considered further in other settings in which parenteral iron is used during pregnancy.

Compared with standard of care, ferric carboxymaltose did not significantly reduce anaemia at the REVAMP trial primary timepoint (36 weeks of gestation) or at delivery, but we now find that ferric carboxymaltose reduced anaemia up to 6 months postpartum, and reduced iron deficiency and iron deficiency anaemia at all timepoints up to 12 months postpartum. The effect on anaemia was driven by the women who were iron deficient at baseline. Post-delivery, women were not given oral iron supplements through the trial programme. The REVAMP trial had initially recruited women with moderate or severe anaemia and no peripheral blood evidence of *Plasmodium* spp infection by rapid diagnostic test; these were judged to be feasible inclusion criteria that could be translated to policy. Determinants of anaemia during pregnancy in this setting were complex (eg, iron deficiency, inflammation, and subpatent parasitaemia), of which only iron deficiency anaemia was directly addressed by the study intervention. Low-cost rapid diagnostic tests for iron parameters such as ferritin are not currently available, which prevented us from basing eligibility for the trial on iron deficiency. A possible driver of inflammation in this population might have been sub-patent *Plasmodium* spp parasitaemia, which might have red cell-specific effects beyond functional iron deficiency and might be refractory to iron therapy. During childbirth, women undergo blood loss, which further diminishes iron stores; the increase in iron stores from ferric carboxymaltose might have provided them with protection against iron deficiency anaemia and anaemia overall as they entered the postpartum period.

To our knowledge, the present analysis is the first long-term postpartum follow-up of mothers and their babies receiving intravenous iron and provides crucial new data on the longevity of the impact on maternal iron stores as well as new insights into the transfer of iron to the infant, building on established evidence for very early superiority for intravenous iron in improving antenatal haemoglobin concentrations.^28^ We were able to leverage REVAMP's randomised controlled experimental design. Although REVAMP was, by necessity of feasibility in the site, open label, our measurements are laboratory parameters, measured and documented distant from the participant and nurse collecting the sample in a local (haemoglobin) or international (ferritin) laboratory, with correspondingly lower risk of bias. Our study experienced loss to follow-up for blood collection at various timepoints for infants; this was likely to be associated with parental concerns regarding blood collection and the difficulty of collection of adequate samples for serum analysis in this age group. However, our comparison of baseline data between included and the overall infant sample indicate a representative sample of the population, and the effect size estimates indicate that it is unlikely that a clinically important effect has been missed. One limitation of the study is that it was powered to detect differences in anaemia and birthweight at 36 weeks of gestation and delivery, respectively. However, the extended follow-up study provides useful information on the plausible range of the effect of intravenous iron compared with standard of care postpartum due to the rigorous design of the original individually randomised trial and the repeated and the concurrent measurements of maternal and infant biochemical and haematological outcomes during the first year of life.

In women in Malawi with moderate or severe anaemia, a single dose of ferric carboxymaltose in the second trimester produced a sustained benefit on maternal iron stores and reduces postpartum anaemia without influencing iron stores or haemoglobin concentrations in their infants.

### Contributors

### Equitable partnership declaration

### Data sharing

Underlying de-identified individual participant data encompassing the reported trial results and a data dictionary are accessible at figshare. Data are available under the terms of Creative Commons Attribution 4.0 International License (CC-BY 4.0).

## Declaration of interests

S-RP reports grants to his institution from the Australian National Health and Medical Research Council and the Bill & Melinda Gates Foundation, providing salary and research support; paid advisory board roles for CSL-Vifor for iron and immunity and for vamifeport in sickle cell disease; consultancy for ITL Biomedical on point-of-care devices in iron; research support from WHO; and unpaid roles as Director (WHO Collaborating Centre for Anaemia Detection and Control) and as co-Chair (Guideline Development Group Meetings). RA owns stock in CSL-Vifor (manufacturer of ferric carboxymaltose). All other authors declare no competing interests.

## Supplementary Materials

Chichewa translation of the abstract

Supplementary appendix 2

Equitable Partnership Declaration

## References

[1] Stevens GA, Paciorek CJ, Flores-Urrutia MC (2022). National, regional, and global estimates of anaemia by severity in women and children for 2000–19: a pooled analysis of population-representative data. Lancet Glob Health.

[2] WHO (2012).

[3] Ba DM, Ssentongo P, Kjerulff KH (2019). Adherence to iron supplementation in 22 sub-Saharan African countries and associated factors among pregnant women: a large population-based study. Curr Dev Nutr.

[4] Milman N (2012). Postpartum anemia II: prevention and treatment. Ann Hematol.

[5] Moya E, Phiri N, Choko AT, Mwangi MN, Phiri KS (2022). Effect of postpartum anaemia on maternal health-related quality of life: a systematic review and meta-analysis. BMC Public Health.

[6] Achebe MM, Gafter-Gvili A (2017). How I treat anemia in pregnancy: iron, cobalamin, and folate. Blood.

[7] Pavord S, Daru J, Prasannan N (2020). UK guidelines on the management of iron deficiency in pregnancy. Br J Haematol.

[8] Pasricha SR, Mwangi MN, Moya E (2023). Ferric carboxymaltose versus standard-of-care oral iron to treat second-trimester anaemia in Malawian pregnant women: a randomised controlled trial. Lancet.

[9] Davidson EM, Simpson JA, Fowkes FJI (2023). The interplay between maternal–infant anemia and iron deficiency. Nutr Rev.

[10] Fisher AL, Nemeth E (2017). Iron homeostasis during pregnancy. Am J Clin Nutr.

[11] Harding R, Ataide R, Mwangi MN (2022). A Randomized controlled trial of the Effect of intraVenous iron on Anaemia in Malawian Pregnant women (REVAMP): statistical analysis plan. Gates Open Res.

[12] Braat S, Fielding KL, Han J (2024). Haemoglobin thresholds to define anaemia from age 6 months to 65 years: estimates from international data source. Lancet Haematol.

[13] WHO (2024).

[14] Pasricha SR, Rogers L, Branca F, Garcia-Casal MN (2024). Measuring haemoglobin concentration to define anaemia: WHO guidelines. Lancet.

[15] Pasricha SR, Hasan MI, Braat S (2021). Benefits and risks of iron interventions in infants in rural Bangladesh. N Engl J Med.

[16] WHO (2020).

[17] Zou G (2004). A modified Poisson regression approach to prospective studies with binary data. Am J Epidemiol.

[18] Liang K-Y, Zeger SL (2000). Longitudinal data analysis of continuous and discrete responses for pre-post designs. Sankhya Ser B.

[19] Moya E, Mzembe G, Mwambinga M (2023). Prevalence of early postpartum depression and associated risk factors among selected women in southern Malawi: a nested observational study. BMC Pregnancy Childbirth.

[20] Seid MH, Derman RJ, Baker JB, Banach W, Goldberg C, Rogers R (2008). Ferric carboxymaltose injection in the treatment of postpartum iron deficiency anemia: a randomized controlled clinical trial. Am J Obstet Gynecol.

[21] Muñoz M, Peña-Rosas JP, Robinson S (2018). Patient blood management in obstetrics: management of anaemia and haematinic deficiencies in pregnancy and in the post-partum period: NATA consensus statement. Transfus Med.

[22] Ataide R, Fielding K, Pasricha SR, Bennett C (2023). Iron deficiency, pregnancy, and neonatal development. Int J Gynaecol Obstet.

[23] Awomolo AM, McWhirter A, Sadler LC, Coppola LM, Hill MG (2023). Intravenous infusions of ferumoxytol compared to oral ferrous sulfate for the treatment of anemia in pregnancy: a randomized controlled trial. Am J Obstet Gynecol MFM.

[24] Awomolo AM, McWhirter A, Sadler LC, Coppola LM, Hill MG (2023). Neonatal outcomes from a randomized controlled trial of maternal treatment of iron deficiency anemia with intravenous ferumoxytol vs oral ferrous sulfate. Am J Obstet Gynecol MFM.

[25] Braithwaite VS, Mwangi MN, Jones KS (2021). Antenatal iron supplementation, FGF23, and bone metabolism in Kenyan women and their offspring: secondary analysis of a randomized controlled trial. Am J Clin Nutr.

[26] Sangkhae V, Fisher AL, Wong S (2020). Effects of maternal iron status on placental and fetal iron homeostasis. J Clin Invest.

[27] Sangkhae V, Fisher AL, Chua KJ, Ruchala P, Ganz T, Nemeth E (2020). Maternal hepcidin determines embryo iron homeostasis in mice. Blood.

[28] Rogozińska E, Daru J, Nicolaides M (2021). Iron preparations for women of reproductive age with iron deficiency anaemia in pregnancy (FRIDA): a systematic review and network meta-analysis. Lancet Haematol.

