# Supplementary material for: Postpartum maternal and infant haematological effects of second-trimester ferric carboxymaltose versus standard-of-care oral iron in Malawi: longitudinal follow-up of a randomised controlled trial
Source: Lancet Glob Health. 2024 Nov 20;12(12):e2049–58. doi: 10.1016/S2214-109X(24)00380-2 (PMC11584314; doi:10.1016/S2214-109X(24)00380-2)
Supplement: Chichewa translation of the abstract [file mmc1.pdf]

THE LANCET
Global Health

Supplementary appendix 1

This translation in Chichewa was submitted by the authors and we reproduce it as supplied. It has not been peer reviewed. *The Lancet*'s editorial processes have only been applied to the original in English, which should serve as reference for this manuscript.

Kutanthauzila kwa mu Chichewa uku kwachokela kwa alembi a nkhaniyi ndipo tayipeleka kwa inu m'mene tinayilandilila. Mawu a mu Chichewa sanawunikilidwenso kapena kukonzedwa. Nkhani ya mu Chingelezi yokha ndi imene yadutsa mu ukonzi wa Lancet, kotelo kuti nkhani ya mu Chingeleziyi ndi imene ikuyimilila mokwanila nkhani yonse imene yalembedwa.

Supplement to: Mzembe G, Moya E, Mwangi MN, et al. Postpartum maternal and infant haematological effects of second-trimester ferric carboxymaltose versus standard-of-care oral iron in Malawi: longitudinal follow-up of a randomised controlled trial. *Lancet Glob Health* 2024; **12:** e2049–58.

Mwachidule

Mau oyamba: Kuchepa kwa magazi ndi kochuluka mwa amayi ndi ana an'gono mu chaka choyamba mai akabereka, makamaka mu maiko a ku Sub-Sahara ku Africa. Tinaunika ngati kupereka chithandizo cha kuchepa kwa magazi pamene mayi ali mu ndime yachiwiri yoyembekezera operekedwa mu msempha a ferric carboxymaltose, posiyanitsa ndi mankhwala amene amayi oyembekezera amalandira nthawi zonse akumwa, ndi cholinga chofuna kuona ngati zingathandizire kuchepetsa vuto la kuchepa kwa magazi mwa amayi amene abereka kumene ndi ana awo.

Njira: REVAMP (ACTRN12618001268235), ndi kafukufuku amene otenga nawo mbali komanso ochititsa kafukufuku amadziwa chithandizo cha mankhwala amene akulandira, komanso otenga nawo mbali amaikidwa mu gulu lolandira mankhwala mwamwayi, ndi cholinga chosiyanitsa zotsatira za magulu awiri olandilira mankhwala owonjezera magazi amumsempha ndi akumwa amene anachitika mu zipatala zisanu ndi zinayi (9) za ku madera a kumudzi, ndi zipatala zisanu (5) za kumadera a ku tauni mu Malawi muno. Kafukufukuyi analemba amayi amene anali mu gawo lachiwiri la pakati amene anali ndi magazi ochepera 10.0gdL atayezedwa mlingo wa magazi, ndipo sanapezeke ndi malungo atayezedwa. Amayi akalembedwa mu kafukufukuyu amagawidwa m'magulu awiri mwamwayi (1:1) kuti alandire mankhwala oonjezera magazi a mu msempha a intravenous ferric carboxymaltose (kuyambira 20 mg/kg kufikira 1000 mg) kapena kulandira mankwala oonjezera magazi akumwa amene azimayi oyembekezera amalandira nthawi zonse (mapilisi a 60 mg okumwa kawiri patsiku kwa masiku 90). Amayi onse otenga nawo mbali mukafukufukuyu analandira mankhwala oteteza ku matenda amalungo. Cholinga cha kafukufuku wa REVAMP chinali kuona kuchuluka kwa mlingo wa magazi pa masabata makumi atatu, asanu ndi imodzi (36) kapena mwezi womaliza wa pakati ndi kuchita kalondolondo wa amayi ndi ana kwa mwezi umodzi mwana akabadwa.

Mu kafukufuku oonjezera wa REVAMP-EXTENDED, amayi ochokera ku kafukufuku wa REVAMP amene anapereka chilolezo, komanso chilolezo cha ana awo, anali pa kalondolondo kwa chaka kuyambira nthawi imene mwana anabadwa ndipo anaonedwa ndi ochita kafukufuku pa miyezi ya 3, 6, 9 ndi 12 atabereka omwe anatenga magazi pa mtsempha kuti akayeze mlingo wa magazi komanso kuchuluka kwa mchere wa ferritin ndi C-reactive protein. Zotsatira za mayi atabereka zinali kuchuluka kwa magazi mthupi (magazi otenga pa mtsempha kuposera 11gdL pa nthawi yobereka komanso magazi oposera 12.0gdL atabereka) kuphatikizirapo kuchuluka kwa magazi ndi mchere wa iron (kuchepa kwa iron kumene kumayezedwa ndi mlingo wa serum ferrin wochepera 15 µg/L, kapena wochepera 30 µg/L ngati mlingo wa mchere wa C-reactive protein uli woposera 5 mg/L, ndi kuchepa kwamagazi kolingana ndi kuchepa kwa iron [kuchepa kwa iron ndi magazi]. Zotsatira za mwana zinai kuchuluka kwa ferritin wochokera ku mchombo, komanso kuchuluka kwa magazi ndi ferrtin pa mwezi woyamba (1), wachitatu (3), wachisanu ndi chimodzi (6), wachisanu ndi chinayi (9) ndi miyezi khumi ndi iwiri (12) kapena chaka.

Zotsatira: Pakati pa 12 Novembala 2018 ndi 2 Malichi 2021, amayi 862 anasankhidwa ndikuikidwa mu gulu limodzi lamagulu awiri a mankhwala operekedwa mumsempha kapena akumwa mwa mwayi mu kafukufuku wa REVAMP. Mwa amayiwa, 793 (393 amena anali mu gulu lolandira mankhwala operekedwa mumsempha a ferric carboxymaltose [ana anabadwa a moyo 376] ndi 400 [ana anabadwa a moyo 376] omwe anali mu gulu lolandira mankhwala akumwa amene amayi oyembekezera amalandira nthawi zonse, anapereka chilolezo chotenga nawo mbali mu kafukufuku woonjezera wa REVAMP-EXTENDED. Pa mwezi wa 12 chiberekereni, ferritin anali wokwererapo (kutengera masamu 1.47[95%CI 1·29–1·66], $p<0·0001$), ndinso chiwerengero cha anthu amene anali ndi mchere wa iron wotsika chinali chochepa (mlingo wotsika 0.65 [0·48–0·88], $p=0·0050$), mwa amayi amene analandira mankhwala a mumsempha a ferric carboxymaltose kusiyana ndi amene analandira mankhwala akumwa amene amayi oyembekezera amalandira nthawi zonse. Kuchepa kwa magazi kunali kocheperako mwa amayi amene analandira mankhawala a mumsempha a ferric carboxymaltose kusiyana ndi mwa amayi amene analandira mankhwala akumwa pa mwezi woyamba atabereka (kusakhala ndi vuto la kuchepa kwa magazi kunali pa 0·84 [95% CI 0·71–0·98], $p=0·027$), pa mwezi wa chitatu atabereka (0·75 [0·62–0·91], $p=0·0029$), komanso pa mwezi wa chisanu ndi chimodzi atabereka (6) (0·78 [0·63–0·96], $p=0·018$) koma izi sizinali chooncho pa miyezi ina yoposera pamenepa. Panalibe umboni wosiyana pakati pa magulu potengera mlingo wa mchere wa ferrtin wa ku mchombo kwa mwana, mlingo wa mchere wa ferritin wa mwana, kapena kuchuluka kwa magazi panthawi ina iliyonse. Vuto la kuchepa magazi kwa amayi amene abereka kumene amene analandira mankhwala a mumsempha linacheperako koposa pakati pa amayi amene anali ndi mlingo wa mchere wa iron wochepa pachiyambi.

Tanthauzo la zotsatira: Chithandizo cha mankhwala owonjezera magazi opereka mu msempha a Ferric carboxymaltose operekedwa mu ndime yachiwiri yapathupi chinateteza ku vuto lochepa magazi kwa amayi obereka kumene komanso kuchepa kwa mchere wa iron mthupi koma sikunakhudze kuchuluka kwa magazi kapena mchere wa iron wa ana.

Wopereka thandizo la ndalama Bill & Melinda Gates Foundation.
