# Supplementary material for: Postpartum maternal and infant haematological effects of second-trimester ferric carboxymaltose versus standard-of-care oral iron in Malawi: longitudinal follow-up of a randomised controlled trial
Source: Lancet Glob Health. 2024 Nov 20;12(12):e2049–58. doi: 10.1016/S2214-109X(24)00380-2 (PMC11584314; doi:10.1016/S2214-109X(24)00380-2)
Supplement: Supplementary appendix 2 [file mmc2.pdf]

THE LANCET
Global Health

Supplementary appendix 2

This appendix formed part of the original submission and has been peer reviewed. We post it as supplied by the authors.

Supplement to: Mzembe G, Moya E, Mwangi MN, et al. Postpartum maternal and infant haematological effects of second-trimester ferric carboxymaltose versus standard-of-care oral iron in Malawi: longitudinal follow-up of a randomised controlled trial. *Lancet Glob Health* 2024; **12:** e2049–58.

Postpartum maternal and infant haematologic effects of antenatal second trimester ferric carboxymaltose compared to standard-of-care oral iron: longitudinal follow-up of a randomised controlled trial in Malawi

Supplemental Appendix

Contents

	Page
- Supplemental Figure 1: Participation in the study	2
- Supplemental Table 1: Haemoglobin and Ferritin concentration availability by timepoint in REVAMP-EXTENDED	3
- Supplemental Table 2: Comparison of baseline characteristics between participating infants contributing to the REVAMP-EXTENDED analyses	5
- Supplemental Table 3: Comparison of baseline characteristics between participating women of the REVAMP-EXTENDED and REVAMP-MAIN	9
- Supplemental Table 4: Infant breastfeeding status	12
- Supplemental Table 5: Maternal and infant outcomes by maternal iron deficiency status – Analysis set	13

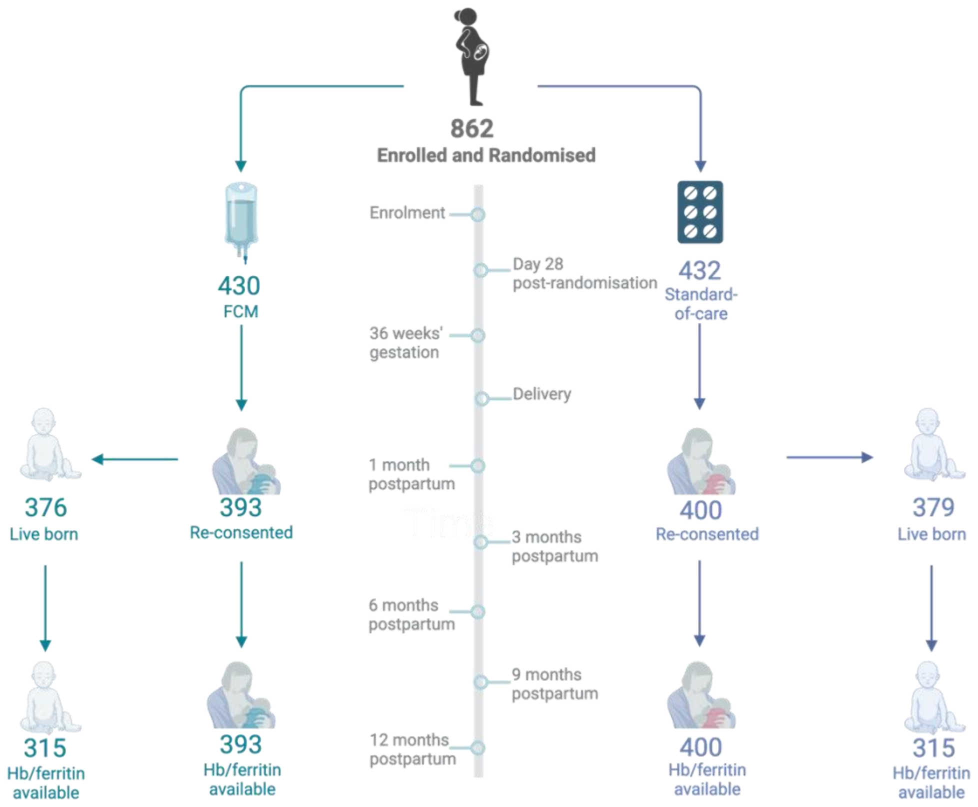

Supplemental Figure 1. Participation in the study

FCM denotes ferric carboxymaltose. Hb denotes Haemoglobin. REVAMP Women were enrolled and randomised into the REVAMP (RandomisEd trial of intraVenous iron for Anaemia in Malawian Pregnant women) open label, individually randomized controlled trial run in Zomba and Blantyre, southern Malawi. Participants were initially followed-up at scheduled visits at 28 days post-enrolment, 36 weeks gestation, during delivery, and 1 month postpartum/of age. Further follow up visits were conducted on retained study participants at 3, 6, 9 and 12 months postpartum/of age. Participants included in this follow up study had been either consented for the full duration of the trial at baseline or consented until 1 month postpartum then re-consented for ongoing follow up after 1 month postpartum. As not all participants gave extended consent, the analyses dataset for all timepoints of the longitudinal follow up study is a subset of the main trial. Availability of laboratory data is defined as having at least one venous Hb or serum ferritin value from enrolment to 12 months postpartum (women) or 1 month to 12 months postpartum (children). Figure created with BioRender.com.

Supplemental Table 1. Haemoglobin and ferritin concentration availability by timepoint in REVAMP-EXTENDED

	Ferric carboxymaltose	Standard of care	P-value*
**Participating mothers†**	N = 393	N=400	
At least one haemoglobin available	393 (100%)	400 (100%)	-
Haemoglobin available by visit			
Enrolment	389 (99%)	400 (100%)	-
28 days post randomisation	366 (93%)	367 (92%)	0·46
36 weeks' gestation	319 (81%)	312 (78%)	0·27
Delivery	343 (87%)	339 (85%)	0·31
1 month postpartum	306 (78%)	307 (77%)	0·71
3 months postpartum	250 (64%)	243 (61%)	0·41
6 months postpartum	290 (74%)	278 (70%)	0·18
9 months postpartum	275 (70%)	262 (66%)	0·18
12 months postpartum	293 (75%)	270 (68%)	0·029
At least one serum ferritin available	393 (100%)	399 (100%)	0·32
Serum ferritin available by visit			
Enrolment	382 (97%)	393 (98%)	-
28 days post randomisation	365 (93%)	360 (90%)	0·15
36 weeks' gestation	307 (78%)	310 (78%)	0·83
Delivery	355 (90%)	359 (90%)	0·78
1 month postpartum	286 (73%)	298 (74%)	0·58
3 months postpartum	240 (61%)	250 (62%)	0·68
6 months postpartum	267 (68%)	252 (63%)	0·14
9 months postpartum	260 (66%)	260 (65%)	0·73
12 months postpartum	257 (65%)	255 (64%)	0·63
**Participating infants‡**	N = 376	N = 379	
At least one haemoglobin available	303 (81%)	302 (80%)	0·76
Haemoglobin available by visit			
1 month	207 (55%)	202 (53%)	0·63
3 months	170 (45%)	151 (40%)	0·14
6 months	180 (48%)	170 (45%)	0·41
9 months	178 (47%)	156 (41%)	0·087
12 months	199 (53%)	183 (48%)	0·20
At least one serum ferritin available	260 (69%)	265 (70%)	0·82
Serum ferritin available by visit			
1 month	143 (38%)	143 (38%)	0·93
3 months	102 (27%)	84 (22%)	0·11
6 months	106 (28%)	108 (28%)	0·93
9 months	94 (25%)	71 (19%)	0·037
12 months	95 (25%)	85 (22%)	0·36
Cord serum ferritin available	280 (74%)	289 (76%)	0·57

n (%) are counts and percentages.

Haemoglobin was obtained using a venous blood sample.

†Participating mothers had been either consented for the full duration of the trial at baseline or consented until 1 month postpartum then re-consented for ongoing follow up after 1 month postpartum.
‡ Participating infants consist of liveborn children whose mothers were randomised and (re)consented for the extended follow up of the trial to 12 months postpartum.
* Avalibility of samples in the ferric carboxymaltose group have compared to the standard-of-care group using a Pearson's chi-squared test. The P-values presented have not been adjusted for multiple comparisons.

Supplemental Table 2. Comparison of baseline characteristics between participating infants contributing to the REVAMP-EXTENDED analyses

	Infants*<br>N = 755	Infants with cord serum ferritin<br>N = 569	Infants with at least one venous haemoglobin<br>N = 605	Infants with at least one serum ferritin<br>N = 525
**Maternal characteristics at enrolment**				
Age (years)	22·3 ± 6·3	22·5 ± 6·5	22·1 ± 6·2	22·4 ± 6·4
Primiparous – n/N (%)‡	412/755 (55%)	308/569 (54%)	337/605 (56%)	283/525 (54%)
Primigravid – n/N (%)‡	408/755 (54%)	305/569 (54%)	334/605 (55%)	281/525 (54%)
Gestational age – weeks				
Median	22·0	22·0	21·9	22·0
Interquartile range (25th to 75th percentile)	19·6-24·3	19·6-24·3	19·6-24·0	19·6-24·3
Height (cm)	155·3 ± 6·4	155·4 ± 6·2	155·2 ± 6·3	155·2 ± 6·3
Weight (kg)	55·5 ± 8·1	55·7 ± 8·3	55·3 ± 7·6	55·7 ± 8·0
Body mass index (kg/m^2)†	23·0 ± 2·9	23·0 ± 3·0	22·9 ± 2·7	23·1 ± 2·9
Religion –n/N (%)‡				
None	2/752 (<1%)	1/567 (<1%)	2/602 (<1%)	1/523 (<1%)
Christian	535/752 (71%)	402/567 (71%)	427/602 (71%)	381/523 (73%)
Muslim	206/752 (27%)	159/567 (28%)	168/602 (28%)	137/523 (26%)
Other	9/752 (1%)	5/567 (<1%)	5/602 (<1%)	4/523 (<1%)
Education –n/N (%)‡				
None	2/727 (<1%)	2/547 (<1%)	2/582 (<1%)	2/506 (<1%)
Lower Primary	155/727 (21%)	114/547 (21%)	127/582 (22%)	102/506 (20%)
Upper Primary	309/727 (43%)	232/547 (42%)	248/582 (43%)	220/506 (43%)
Lower Secondary	103/727 (14%)	82/547 (15%)	84/582 (14%)	81/506 (16%)
Upper Secondary	143/727 (20%)	106/547 (19%)	109/582 (19%)	92/506 (18%)
Tertiary	15/727 (2%)	11/547 (2%)	12/582 (2%)	9/506 (2%)
Marital status –n/N (%)‡				
Single	124/752 (16%)	89/567 (16%)	106/602 (18%)	86/523 (16%)

	Infants*<br><br><br>N = 755	Infants with<br>cord serum ferritin<br><br>N = 569	Infants with<br>at least one venous<br>haemoglobin<br>N = 605	Infants with<br>at least one serum<br>ferritin<br>N = 525
Married	613/752 (82%)	469/567 (83%)	484/602 (80%)	427/523 (82%)
Widowed	3/752 (<1%)	2/567 (<1%)	3/602 (<1%)	3/523 (<1%)
Divorced/Separated	10/752 (1%)	6/567 (1%)	8/602 (1%)	6/523 (1%)
Other	2/752 (<1%)	1/567 (<1%)	1/602 (<1%)	1/523 (<1%)
Income source –n/N (%)‡				
None	49/752 (7%)	39/567 (7%)	43/602 (7%)	34/523 (7%)
Subsistence farming	142/752 (19%)	106/567 (19%)	121/602 (20%)	103/523 (20%)
Large scale farming	2/752 (<1%)	2/567 (<1%)	0/602 (0%)	0/523 (0%)
Employed	122/752 (16%)	96/567 (17%)	89/602 (15%)	77/523 (15%)
Casual work for wages	235/752 (31%)	177/567 (31%)	194/602 (32%)	168/523 (32%)
Business	193/752 (26%)	142/567 (25%)	151/602 (25%)	138/523 (26%)
Other	9/752 (1%)	5/567 (<1%)	4/602 (<1%)	3/523 (<1%)
Re-screened post positive malaria RDT – n/N (%)§	230/755 (30%)	171/569 (30%)	196/605 (32%)	182/525 (35%)
Malaria RDT positive –n/N (%)¶	13/740 (2%)	8/559 (1%)	10/591 (2%)	10/515 (2%)
HIV positive –n/N (%)‡	125/749 (17%)	91/563 (16%)	102/600 (17%)	91/522 (17%)
Capillary Hb<10g/dL –n/N (%)	753/753 (100%)	567/567 (100%)	603/603 (100%)	524/524 (100%)
Venous Hb – g/dL‖	8·8 ± 1·2	8·8 ± 1·2	8·8 ± 1·2	8·8 ± 1·2
Anaemia (based on venous Hb) –n/N (%)				
No (Hb≥11g/dL)	33/751 (4%)	26/565 (5%)	25/603 (4%)	20/524 (4%)
Mild (10g/dL≤Hb<11g/dL)	84/751 (11%)	63/565 (11%)	64/603 (11%)	55/524 (10%)
Moderate (17g/dL≤Hb<10g/dL)	583/751 (78%)	435/565 (77%)	468/603 (78%)	410/524 (78%)
Severe (Hb<7g/dL)	51/751 (7%)	41/565 (7%)	46/603 (8%)	39/524 (7%)
Serum ferritin – µg/L**				
Median	27·4	27·5	28·8	27·8
Interquartile range (25th to 75th percentile)	10·3-72·7	9·7-67·5	10·4-78·0	10·4-76·5

	Infants*<br><br><br>N = 755	Infants with<br>cord serum ferritin<br><br>N = 569	Infants with<br>at least one venous haemoglobin<br>N = 605	Infants with<br>at least one serum ferritin<br>N = 525
C-reactive protein – mg/L**				
Median	5·2	5·2	5·2	5·2
Interquartile range (25th to 75th percentile)	2·8-10·7	2·7-11·1	2·7-10·3	2·7-11·0
Iron deficient –n/N (%)††	313/738 (42%)	240/561 (43%)	241/589 (41%)	217/512 (42%)
Iron deficiency anaemia –n/N (%)††	297/734 (40%)	227/557 (41%)	229/587 (39%)	207/511 (41%)
Inflammation –n/N (%)‡‡	376/738 (51%)	292/561 (52%)	301/589 (51%)	267/512 (52%)
Anaemia and inflammation –n/N (%)‡‡	358/734 (49%)	275/557 (49%)	285/587 (49%)	252/511 (49%)
**Neonate characteristics at delivery**				
Birth weight (grams)§§	2892·9 ± 508·1	2924·8 ± 478·3	2896·4 ± 466·1	2897·6 ± 460·1
Birth length (centimetres)¶¶	47·5 ± 3·6	47·7 ± 3·4	47·5 ± 3·5	47·5 ± 3·5
Sex				
Female	356/736 (48%)	270/566 (48%)	295/603 (49%)	258/523 (49%)
Male	380/736 (52%)	296/566 (52%)	308/603 (51%)	265/523 (51%)
Gestation duration – weeks||||				
Median	39·9	39·9	39·9	39·7
Interquartile range (25th to 75th percentile)	(38·6-40·8)	(38·6-40·7)	(38·6-40·7)	(38·6-40·7)

n/N (%) are counts and percentages Plus-minus values are mean ±SD. SD denotes Standard Deviation, RDT rapid diagnostic test (for *Plasmodium* parasitaemia), Hb haemoglobin, and HIV human immunodeficiency virus.
* Restricted to liveborn children whose mothers were randomised and (re)consented for the extended follow up of the trial to 12 months post-partum. Participants were eligible for the trial if they had a capillary haemoglobin concentration below 10·0 g/dL (moderate or severe anaemia) measured by HemoCue 301+ (HemoCue AB, Angelholm, Sweden). However, trial outcomes of anaemia followed WHO standards of hemoglobin concentration measured by venous blood.
† Body mass index is the weight in kilograms divided by the square of the height in meters.
‡ Religion, Education, Marital status, Income source, parity, gravidity and HIV status were self reported.
§ If women met the anaemia criteria but had a positive RDT, they were treated for malaria as per local protocols and deferred from enrolment. These women were able to present for re-screening – no earlier than seven days later – and be enrolled if they met the eligibility criteria (in these cases, parasitemia was assessed using microscopy due to persistence of antigen detection via RDT).

¶ Malaria RDT positive based on confirmatory RDT testing by lab personal on venous blood collected at enrolment.
|| Data are missing for 4 participants in the ferric carboxymaltose group and 0 participants in the standard-of-care group.
** Data are missing for 11 participants in the ferric carboxymaltose group and 7 participants in the standard-of-care group.
†† Iron deficient indicates serum ferritin<15µg/L or ferritin<30µg/L if C-reactive protein>5mg/L, and iron deficiency anaemia indicates venous Hb<11g/dL and serum ferritin<15µg/L or ferritin<30µg/L if C-reactive protein>5mg/L.
‡‡ Inflammation indicates C-reactive protein>5mg/L, and anaemia and inflammation indicates venous Hb<11·0g/dL and C-reactive protein>5mg/L.
§§ Data are missing for 23 infants and 5, 6, and 6 infants of those with cord ferritin, at least one Hb, and at least one ferritin, respectively.
¶¶ Data are missing for 35 infants and 10, 17, and 15 infants of those with cord ferritin, at least one Hb, and at least one ferritin, respectively.
|||| Data are missing for 15 infants and 0, 0, and 0 infants of those with cord ferritin, at least one Hb, and at least one ferritin, respectively.

Supplemental Table 3. Comparison of baseline characteristics between participating women of the REVAMP-EXTENDED and REVAMP-MAIN

	REVAMP-EXTENDED participants<br><br>N = 793	REVAMP-MAIN participants not in EXTENDED<br><br>N = 69	REVAMP-MAIN participants<br><br>N = 862
**Maternal characteristics at enrolment**			
Age (years)	22·4 ± 6·3	22·4 ± 4·3	22·4 ± 6·2
Primiparous – n/N (%)‡	431/793 (54%)	41/69 (59%)	472/862 (55%)
Primigravid – n/N (%)‡	425/793 (54%)	41/69 (59%)	466/862 (54%)
Gestational age – weeks			
Median	22·0	20·9	21·9
Interquartile range (25th to 75th percentile)	19·4-24·3	19·1-23·3	19·3-24·1
Height (cm)	155·3 ± 6·4	155·7 ± 5·5	155·3 ± 6·3
Weight (kg)	55·5 ± 8·3	57·9 ± 7·7	55·7 ± 8·3
Body mass index (kg/m^2)†	23·0 ± 3·1	23·8 ± 2·7	23·1 ± 3·0
Religion –n/N (%)‡			
None	2/790 (<1%)	0/69 (0%)	2/859 (<1%)
Christian	563/790 (71%)	54/69 (78%)	617/859 (72%)
Muslim	217/790 (27%)	14/69 (20%)	231/859 (27%)
Other	8/790 (1%)	1/69 (1%)	9/859 (1%)
Education –n/N (%)‡			
None	2/763 (<1%)	0/67 (0%)	2/830 (<1%)
Lower Primary	165/763 (22%)	10/67 (15%)	175/830 (21%)
Upper Primary	322/763 (42%)	18/67 (27%)	340/830 (41%)
Lower Secondary	105/763 (14%)	10/67 (15%)	115/830 (14%)
Upper Secondary	151/763 (20%)	27/67 (40%)	178/830 (21%)
Tertiary	18/763 (2%)	2/67 (3%)	20/830 (2%)
Marital status –n/N (%)‡			
Single	126/790 (16%)	8/69 (12%)	134/859 (16%)
Married	649/790 (82%)	60/69 (87%)	709/859 (83%)
Widowed	3/790 (<1%)	1/69 (1%)	4/859 (<1%)
Divorced/Separated	10/790 (1%)	0/69 (0%)	10/859 (1%)
Other	2/790 (<1%)	0/69 (0%)	2/859 (<1%)
Income source –n/N (%)‡			
None	51/790 (6%)	2/69 (3%)	53/859 (6%)
Subsistence farming	149/790 (19%)	2/69 (3%)	151/859 (18%)
Large scale farming	2/790 (<1%)	0/69 (0%)	2/859 (<1%)

	REVAMP-EXTENDED participants<br><br>N = 793	REVAMP-MAIN participants not in EXTENDED<br><br>N = 69	REVAMP-MAIN participants<br><br>N = 862
Employed	126/790 (16%)	27/69 (39%)	153/859 (18%)
Casual work for wages	249/790 (32%)	15/69 (22%)	264/859 (31%)
Business	202/790 (26%)	22/69 (32%)	224/859 (26%)
Other	11/790 (1%)	1/69 (1%)	12/859 (1%)
Re-screened post positive malaria RDT –n/N (%)§	238/793 (30%)	16/69 (23%)	254/862 (29%)
Malaria RDT positive –n/N (%)¶	12/777 (2%)	12/69 (17%)	12/846 (1%)
HIV positive –n/N (%)‡	133/786 (17%)	0/69 (0%)	145/855 (17%)
Capillary Hb<10g/dL –n/N (%)	791/791 (100%)	69/69 (100%)	860/860 (100%)
Venous Hb – g/dL||	8·82 ± 1·23	8·98 ± 1·46	8·83 ± 1·25
Anaemia (based on venous Hb) – n/N (%)			
No (Hb≥11g/dL)	35/789 (4%)	7/69 (10%)	42/858 (5%)
Mild (10g/dL≤Hb<11g/dL)	94/789 (12%)	11/69 (16%)	105/858 (12%)
Moderate (17g/dL≤Hb<10g/dL)	605/789 (77%)	44/69 (64%)	649/858 (76%)
Severe (Hb<7g/dL)	55/789 (7%)	7/69 (10%)	62/858 (7%)
Serum ferritin – µg/L**			
Median	27·1	19·0	26·7
Interquartile range (25th to 75th percentile)	10·2 – 73·0	7·5 – 63·6	9·9 – 72·3
C-reactive protein – mg/L**			
Median	5·20	5·30	5·20
Interquartile range (25th to 75th percentile)	2·80 – 11·00	2·30 – 9·60	2·80 – 10·60
Iron deficient –n/N (%)††	328/775 (42%)	38/69 (55%)	366/844 (43%)
Iron deficiency anaemia –n/N (%)††	312/771 (40%)	37/69 (54%)	349/840 (42%)
Inflammation –n/N (%)‡‡	395/775 (51%)	37/69 (54%)	432/844 (51%)
Anaemia and inflammation –n/N (%)‡‡	376/771 (49%)	35/69 (51%)	411/840 (49%)

Participants were enrolled across two sites in REVAMP-EXTENDED, Blantyre (N=95) and Zomba (N=698), and REVAMP-MAIN, Blantyre (N=139) and Zomba (N=723).
n/N (%) are counts and percentages Plus-minus values are mean ±SD. SD denotes Standard Deviation, RDT rapid diagnostic test (for *Plasmodium* parasitaemia), Hb haemoglobin, and HIV human immunodeficiency virus.
*REVAMP-EXTENDED participants had been either consented for the full duration of the trial at baseline or consented until 1 month postpartum then re-consented for ongoing follow up after 1 month postpartum. As not all participants gave extended consent, this is a subset of the REVAMP-MAIN participants. Participants were eligible for the trial if they had a capillary haemoglobin concentration below 10·0 g/dL (moderate or severe anaemia)

measured by HemoCue 301+ (HemoCue AB, Angelholm, Sweden). However, trial outcomes of anaemia followed WHO standards of hemoglobin concentration measured by venous blood.
† Body mass index is the weight in kilograms divided by the square of the height in meters.
‡ Religion, Education, Marital status, Income source, parity, gravidity and HIV status were self reported.
§ If women met the anaemia criteria but had a positive RDT, they were treated for malaria as per local protocols and deferred from enrolment. These women were able to present for re-screening – no earlier than seven days later – and be enrolled if they met the eligibility criteria (in these cases, parasitemia was assessed using microscopy due to persistence of antigen detection via RDT).
¶ Malaria RDT positive based on confirmatory RDT testing by lab personal on venous blood collected at enrolment.
|| Data are missing for 4 participants in the ferric carboxymaltose group and 0 participants in the standard-of-care group.
** Data are missing for 11 participants in the ferric carboxymaltose group and 7 participants in the standard-of-care group.
†† Iron deficient indicates serum ferritin<15µg/L or ferritin<30µg/L if C-reactive protein>5mg/L, and iron deficiency anaemia indicates venous Hb<11g/dL and serum ferritin<15µg/L or ferritin<30µg/L if C-reactive protein>5mg/L.
‡‡ Inflammation indicates C-reactive protein>5mg/L, and anaemia and inflammation indicates venous Hb<11·0g/dL and C-reactive protein>5mg/L.

Supplemental Table 4. Infant breastfeeding status

	Ferric carboxymaltose N = 376	Standard of care N = 379
**Exclusive breastfeeding***		
1 month	219/228 (96%)	233/242 (96%)
3 months	210/240 (88%)	219/250 (88%)
6 months	23/271 (8%)	22/272 (8%)

Data are presented as n/N (%).
*Exclusive breastfeeding reflects the number of infants who were exclusively breastfed up-to and including 1, 3 and 6 months respectively.

Supplemental Table 5. Maternal and infant outcomes by maternal iron deficiency status - Analysis set*

**Maternal outcomes**									
	**Iron deficient at baseline‡**				**Non-iron deficient at baseline‡**				
Visit	Ferric carboxymaltose<br><br>N = 163	Standard of care<br><br>N = 165	Prevalence Ratio§ or Mean difference¶ or Geometric mean ratio|| (95% CI)	P-value**	Ferric carboxymaltose<br><br>N = 219	Standard of care<br><br>N = 228	Prevalence Ratio§ or Mean difference¶ or Geometric mean ratio|| (95% CI)	P-value**	P-value for interaction**
**Anaemia†§**									
1 month postpartum	54/133 (41%)	64/123 (52%)	0·77 (0·59, 1·01)	0·060	80/163 (49%)	99/179 (55%)	0·87 (0·71, 1·07)	0·19	0·48
3 months postpartum	33/98 (34%)	55/93 (59%)	0·56 (0·41, 0·77)	0·0003	63/142 (44%)	70/146 (48%)	0·91 (0·71, 1·16)	0·44	0·019
6 months postpartum	33/116 (28%)	53/113 (47%)	0·61 (0·43, 0·86)	0·0045	61/165 (37%)	64/160 (40%)	0·92 (0·70, 1·21)	0·54	0·066
9 months postpartum	39/114 (34%)	47/106 (44%)	0·78 (0·56, 1·08)	0·13	69/153 (45%)	61/151 (40%)	1·10 (0·85, 1·43)	0·46	0·10
12 months postpartum	48/125 (38%)	42/110 (38%)	0·98 (0·71, 1·36)	0·92	68/159 (43%)	57/155 (37%)	1·17 (0·89, 1·54)	0·25	0·42
**Iron deficient‡§**									
1 month postpartum	25/116 (22%)	49/121 (40%)	0·51 (0·34, 0·78)	0·0015	9/160 (6%)	35/172 (20%)	0·27 (0·13, 0·54)	0·0002	0·12
3 months postpartum	24/97 (25%)	42/102 (41%)	0·53 (0·35, 0·80)	0·0024	4/135 (3%)	20/144 (14%)	0·21 (0·08, 0·59)	0·0030	0·11
6 months postpartum	30/109 (28%)	46/108 (43%)	0·59 (0·40, 0·86)	0·0056	9/154 (6%)	27/141 (19%)	0·30 (0·15, 0·60)	0·0007	0·096
9 months postpartum	35/110 (32%)	50/104 (48%)	0·61 (0·43, 0·85)	0·0041	9/143 (6%)	26/151 (17%)	0·36 (0·18, 0·74)	0·0050	0·20

12 months postpartum	43/116 (37%)	43/106 (41%)	0·82 (0·59, 1·16)	0·26	11/135 (8%)	28/144 (19%)	0·42 (0·22, 0·79)	0·0076	0·068
**Iron deficient anaemia‡§**									
1 month postpartum	17/114 (15%)	31/108 (29%)	0·46 (0·27, 0·79)	0·0052	8/149 (5%)	26/158 (16%)	0·30 (0·14, 0·66)	0·0025	0·39
3 months postpartum	10/90 (11%)	30/88 (34%)	0·27 (0·14, 0·51)	0·0001	1/130 (<1%)	13/134 (10%)	0·08 (0·01, 0·56)	0·011	0·25
6 months postpartum	16/104 (15%)	30/101 (30%)	0·44 (0·26, 0·77)	0·0034	8/150 (5%)	14/135 (10%)	0·48 (0·21, 1·08)	0·076	0·88
9 months postpartum	16/108 (15%)	28/96 (29%)	0·43 (0·25, 0·74)	0·0025	6/138 (4%)	17/138 (12%)	0·34 (0·14, 0·81)	0·015	0·65
12 months postpartum	25/114 (22%)	22/100 (22%)	0·76 (0·45, 1·29)	0·31	9/133 (7%)	16/131 (12%)	0·55 (0·25, 1·18)	0·13	0·49
**Venous haemoglobin (g/dL)†¶**									
1 month postpartum	12·15 ± 1·39	11·80 ± 1·44	0·41 (0·11, 0·72)	0·0083	11·80 ± 1·33	11·73 ± 1·21	0·18 (-0·08, 0·45)	0·18	0·26
3 months postpartum	12·26 ± 1·08	11·73 ± 1·47	0·53 (0·23, 0·84)	0·0005	11·99 ± 1·20	11·94 ± 1·13	0·07 (-0·19, 0·32)	0·61	0·020
6 months postpartum	12·28 ± 1·11	11·95 ± 1·52	0·27 (-0·04, 0·58)	0·087	12·21 ± 1·16	12·16 ± 1·34	0·06 (-0·21, 0·32)	0·67	0·30
9 months postpartum	12·10 ± 1·23	12·08 ± 1·47	-0·05 (-0·35, 0·25)	0·74	12·03 ± 1·13	12·20 ± 1·09	-0·10 (-0·36, 0·15)	0·42	0·79
12 months postpartum	12·06 ± 1·44	12·03 ± 1·49	0·11 (-0·21, 0·43)	0·50	12·15 ± 1·23	12·17 ± 1·23	-0·05 (-0·32, 0·23)	0·74	0·47
**Serum ferritin (µg/L)‡||**									
1 month postpartum	46·1 (19·0-85·8)	23·8 (13·0-51·0)	1·63 (1·32, 2·01)	<0·0001	85·1 (51·5-155·1)	36·3 (17·5-67·0)	2·24 (1·87, 2·67)	<0·0001	0·024
3 months postpartum	38·2 (15·5-74·7)	20·7 (12·9-37·1)	1·66 (1·36, 2·04)	<0·0001	65·60 (43·6-122·6)	31·1 (17·9-50·7)	2·11 (1·77, 2·51)	<0·0001	0·082
6 months postpartum	30·8 (14·8-50·5)	18·8 (9·7-34·7)	1·54 (1·29, 1·84)	<0·0001	65·4 (36·4-115·5)	35·7 (19·7-47·7)	1·91 (1·64, 2·23)	<0·0001	0·075
9 months postpartum	23·2 (12·0-48·1)	16·6 (10·9-28·9)	1·45 (1·21, 1·74)	<0·0001	61·1 (35·6-104·0)	31·3 (19·0-47·2)	1·85 (1·59, 2·16)	<0·0001	0·045

12 months postpartum	20·7 (10·5-44·9)	19·5 (11·8-32·4)	1·25 (1·03, 1·52)	0·022	57·6 (32·8-103·5)	33·1 (18·8-47·7)	1·66 (1·40, 1·97)	<0·0001	0·032
**Infant outcomes**									
	**Iron deficient at baseline‡**				**Non-iron deficient at baseline‡**				
Visit	Ferric carboxymaltose<br><br>N = 158	Standard of care<br><br>N = 155	Mean difference¶ or Geometric mean ratio|| (95% CI)	P-value**	Ferric carboxymaltose<br><br>N = 207	Standard of care<br><br>N = 218	Mean difference¶ or Geometric mean ratio|| (95% CI)	P-value**	P-value for interaction**
**Venous haemoglobin (g/dL)¶**									
1 month	11·79 ± 1·72	11·43 ± 1·83	0·42 (-0·12, 0·97)	0·12	12·04 ± 1·90	12·29 ± 1·79	-0·16 (-0·62, 0·30)	0·49	0·11
3 months	10·52 ± 1·47	10·50 ± 1·17	0·18 (-0·21, 0·57)	0·38	10·20 ± 1·10	10·51 ± 1·05	-0·38 (-0·69, -0·06)	0·018	0·030
6 months	10·30 ± 1·18	10·28 ± 1·11	-0·05 (-0·42, 0·32)	0·79	9·97 ± 1·33	10·16 ± 1·18	-0·16 (-0·47, 0·14)	0·30	0·65
9 months	10·10 ± 1·11	9·85 ± 1·06	0·28 (-0·11, 0·68)	0·16	9·82 ± 1·43	9·86 ± 1·14	-0·09 (-0·41, 0·23)	0·58	0·15
12 months	9·97 ± 1·12	9·99 ± 1·25	-0·03 (-0·40, 0·35)	0·88	9·96 ± 1·31	10·08 ± 1·18	-0·06 (-0·36, 0·25)	0·71	0·90
**Serum ferritin (µg/L)||**									
1 month	206·6 (158·6-324·4)	223·9 (147·9-349·9)	1·01 (0·80, 1·28)	0·91	281·3 (204·4-389·4)	223·8 (171·6-341·9)	0·96 (0·78, 1·18)	0·69	0·73
3 months	90·6 (64·2-143·4)	124·1 (48·6-197·5)	1·01 (0·73, 1·38)	0·97	128·7 (67·4-190·5)	94·0 (61·6-166·3)	1·07 (0·83, 1·38)	0·61	0·77
6 months	19·3 (9·6-34·3)	32·6 (15·5-49·4)	0·85 (0·61, 1·18)	0·32	32·9 (17·8-57·5)	29·3 (18·5-43·5)	0·96 (0·74, 1·23)	0·73	0·57
9 months	17·3 (8·1-32·5)	16·9 (9·9-27·6)	1·05 (0·75, 1·49)	0·76	16·8 (8·3-25·8)	16·8 (9·9-26·6)	0·94 (0·71, 1·25)	0·66	0·61
12 months	11·9 (7·9-18·5)	17·6 (9·3-24·2)	0·69 (0·49, 0·98)	0·036	16·2 (9·8-27·6)	14·7 (8·1-26·2)	0·99 (0·73, 1·33)	0·94	0·13

Data are presented as n/N (%), mean ±SD or median and interquartile range. SD denotes Standard Deviation and CI confidence interval.

* The maternal analysis set is restricted to those randomised women that (re)consented for the extended follow up of the trial to 12 months postpartum and with at least one outcome from entolment to 12 months postpartum, which is 793 women. The infant analysis set is restricted to liveborn children whose mothers were randomised and (re)consented for the extended follow up of the trial to 12 months postpartum and with at least one outcome from 1 month to 12 months of age.
† Anaemia indicates venous haemoglobin<11·0g/dL up to and including delivery and venous haemoglobin<12·0g/dL postpartum. Data are missing for 4 participants in the ferric carboxymaltose group and 0 participants in the standard-of-care group.
‡ Iron deficient indicates serum ferritin<15µg/L or ferritin<30µg/L if C-reactive protein>5mg/L, and iron deficiency anaemia indicates venous haemoglobin<11g/dL and serum ferritin<15µg/L or ferritin<30µg/L if C-reactive protein>5mg/L. Data are missing for 11 participants in the ferric carboxymaltose group and 7 participants in the standard-of-care group.
§ A prevalence ratio of ferric carboxymaltose versus standard of care is displayed for maternal anaemia, iron deficiency, and iron deficiency anaemia at 1, 3, 6, 9 and 12 months postpartum following analyses using a Poisson model with random intercept for the women and robust standard errors. Subgroup (main effect) and subgroup-by-treatment interactions terms have been added to the models to evaluate where the treatment effect (ferric carboxymaltose versus standard of care) differs between subgroup categories for iron deficiency at baseline.
¶An absolute mean difference of ferric carboxymaltose versus standard of care of the estimated change from baseline to 1, 3, 6, 9 and 12 months postpartum is displayed for maternal and infant continuous haemoglobin following analyses using a longitudinal data analysis model. Subgroup (main effect) and subgroup-by-treatment interactions terms have been added to the models to evaluate where the treatment effect (ferric carboxymaltose versus standard of care) differs between subgroup categories for iron deficiency at baseline.
|| A geometric mean ratio is displayed for maternal and infant log-transformed ferritin concentration. The model for maternal outcomes included all timepoints from baseline to 12 months postpartum). Subgroup (main effect) and subgroup-by-treatment interactions terms have been added to the models to evaluate where the treatment effect (ferric carboxymaltose versus standard of care) differs between subgroup categories for iron deficiency at baseline.
** The P-values and 95% confidence intervals presented have not been adjusted for multiple comparisons.
