# Supplementary material for: Postpartum maternal and infant haematological effects of second-trimester ferric carboxymaltose versus standard-of-care oral iron in Malawi: longitudinal follow-up of a randomised controlled trial
Source: Lancet Glob Health. 2024 Nov 20;12(12):e2049–58. doi: 10.1016/S2214-109X(24)00380-2 (PMC11584314; doi:10.1016/S2214-109X(24)00380-2)
Supplement: Equitable Partnership Declaration [file mmc3.pdf]

THE LANCET
Global Health

Supplementary appendix 3

This Equitable Partnership Declaration (EPD) was submitted by the authors, and we reproduce it as supplied. It has not been peer reviewed. *The Lancet*'s editorial processes have not been applied to the EPD.

Supplement to: Mzembe G, Moya E, Mwangi MN, et al. Postpartum maternal and infant haematological effects of second-trimester ferric carboxymaltose versus standard-of-care oral iron in Malawi: longitudinal follow-up of a randomised controlled trial. *Lancet Glob Health* 2024; **12:** e2049–58.

Equitable Partnership Declaration questions

Researcher considerations

1. Please detail the involvement that researchers who are based in the region(s) of study had during a) study design; b) clinical study processes, such as processing blood samples, prescribing medication, or patient recruitment; c) data interpretation; and d) manuscript preparation, commenting on all aspects. If they were not involved in any of these aspects, please explain why.

   This question is intended for international partnerships; if all your authors are based in the area of study, this question is not applicable.

   This should include a thorough description of their leadership role(s) in the study. Are local researchers named in the author list or the acknowledgements, or are they not mentioned at all (and, if not, why)? Please also describe the involvement of early career researchers based in the location of the study. Some of this information might be repeated from the Contributors section in the manuscript. Note: we adhere to [ICMJE authorship criteria] *when deciding who should be named on a paper.*

**a) Study design:** Local researchers took a leading role in the design and implementation of the trial. Prof Kamija S Phiri is a local Co-Principal Investigator and was involved in the idea's conception and fund sourcing. A local team comprising a Post Doctoral Scientist and two local PhD fellows was heavily involved in drafting and developing the trial protocol, the standard operating procedures and the data collection tools for the study.
**b) Clinical study processes:** Local researchers conducted all clinical study procedures, including participant screening and recruitment, trial medication administration, sample collection, and follow-up assessments. The local team also processed and stored samples for all locally available tests.
**c) Data interpretation:** Local researchers were involved in all stages of data cleaning (including query generation and resolution), data analysis and interpretation.
**d) Manuscript preparation:** Local authors were involved in all stages of manuscript preparation and took leading and senior authorship roles.<br><br>We have **bolded** the local researchers on the authorship list below:<br>**Glory Mzembe MSc, Ernest Moya PhD, Martin N Mwangi PhD,** Ricardo Ataide PhD, Rebecca Harding PhD, **Justina Kaunda MSc, Truwah Zinenani BBA, Gomezgani Mhango BSc, Prof William Stones FRCOG, Owen Mtambo PhD**, Ayse Y Demir MD, Hans Verhoef PhD, Sabine Braat MSc, Prof Sant-Rayn Pasricha PhD, **Prof Kamija S Phiri PhD**<br>**Glory Mzembe and Ernest Moya** are early career scientists recruited as PhD fellows on the trial.

2. Were the data used in your study collected by authors named on the paper, or have they been extracted from a source such as a national survey? ie, is this a secondary analysis of data that were not collected by the authors of this paper. If the authors of this paper were not involved in data collection, how were data interpreted with sufficient contextual knowledge?

   The Lancet Global Health *believe contextual understanding is crucial for informed data analysis and interpretation.*

All data presented in the manuscript were collected by the authors named on the paper.

3. How was funding used to remunerate and enhance the skills of researchers and institutions based in the area(s) of study? And how was funding used to improve research infrastructure in the area of study?

   Potentially effective investments into long-term skills and opportunities within institutions could include training or mentorship in analytical techniques and manuscript writing, opportunities to lead all or specific aspects of the study, financial remuneration rather than requiring volunteers, and other professional development and educational opportunities.

   Improvements to research infrastructure could be funding of extended trial designs (such as platform trials) and use of master protocols to enable these designs, establishment of long-term contracts for research staff, building research facilities, and local control of funding allocation.

**Skills:** The study trained two local PhDs, one Postdoc and one master's student. The study also funded in person certified trainings on Good Clinical Practice (GCP), Ultrasound Scan Dating, Emergency Triage Assessment and Treatment (ETAT) and supported training and mentorship in data analysis techniques and manuscript writing. In addition to this, funding permitted the establishment of new sample collection protocols locally, and attendance at workshops and conferences.
**Research infrastructure:** Funding for all data collection activities was controlled locally. The study supported the purchase of laboratory equipment (including equipment for EEG, ABR and low-resolution MRIs - which are not reported in this manuscript) and study vehicles, and contributed towards the building of research facilities which are now a permanent asset for the local institution.

4. How did you safeguard the researchers who implemented the study?

   Please describe how you guaranteed safe working conditions for study staff, including provision of appropriate personal protective equipment, protection from violence, and prevention of overworking.

The study procured insurance (professional indemnity) for all relevant staff and provided appropriate personal protective equipment to all staff. This included a significant adjustment to the levels of appropriate personal protective equipment, hand-wash and cleaning facilities during the Covid era. All local staff were trained on the relevant safety measures and guidelines. Extensive community awareness campaigns were conducted by the local study team before the beginning of the study to ensure the safety of the study staff when conducting field activities. All staff completed time sheets to prevent overworking.

<u>Benefits to the communities and regions of study</u>

5. How does the study address the research and policy priorities of its location?

   How were the local priorities determined and then used to inform the research question? Who decided which priorities to take forward? Which elements of the study address those priorities?

The study addresses anaemia, one of the major public health issues in the study area. Anaemia reduction is included as one of six World Health Assembly Global Nutrition Targets within the

Comprehensive implementation plan on maternal, infant and young child nutrition. Additionally, anaemia in women 15–49 years of age is one of the targets for the United Nations 2030 Agenda for Sustainable Development. Further, reduction of anaemia in vulnerable populations such as pregnant, postpartum women and children under-five is one of Malawi's research agendas on building human capital. The Study Principal Investigators conceived the trial after assessing the high burden of anaemia in the location and sought to directly address the research and policy priorities of the local area. The study, which follows a pragmatic approach, was specifically designed to allow scale up of the intervention in the event of a positive outcome.

6. How will research products be shared in the community of study?

For instance, will you be providing written or oral layperson summaries for non-academic information sharing? Will study data be made available to institutions in the region(s) of study? The Lancet Global Health *encourages authors to translate the summary (abstract) into relevant languages after paper editing; do you intend to translate your summary?*

Both written and oral layperson summaries will be used for non-academic information sharing in the community of the study.

7. How were individuals, communities, and environments protected from harm?

a) How did you ensure that sensitive patient data was handled safely and respectfully? Was there any potential for stigma or discrimination against participants arising from any of the procedures or outcomes of the study?

Participants' IDs were used in all patient files instead of names. All files were kept in lockable stationery cabinets with limited access. Participant names were only indicated on informed consent forms which were kept separately from other participant files and had very limited access even to trial staff. All data collection electronic gadgets i.e. tablets were password-protected and were only used for data collection. Data was moved from the tablets to the server on a daily basis to ensure participant confidentiality in case of loss or theft. There was no potential for stigma or discrimination against participants arising from any of the study procedures or outcomes of the study.

b) Might any of the tests be experienced as invasive or culturally insensitive?

In Malawi, the collection of blood samples in children is usually considered sensitive in the community. We addressed this issue in the many awareness campaigns and the study design for the main trial and the extended study which were designed to allow women to opt out of blood collection from their infants without affecting their continued participation in the trial.

c) How did you determine that work was sensitive to traditions, restrictions, and considerations of all cultural and religious groups in the study population?

As the trial protocol, study design and case report forms were heavily written by local investigators and approved by a local Ethics board, all consideration was given to local traditions, restrictions and cultural or religious concerns. Before the implementation of the study, a series of community engagement activities were conducted with the local communities. This included meeting with traditional leaders, religious representatives and other community representatives, explaining the study objectives and detailing the study procedures to get feedback from them on whether it would be acceptable by the community or if there are components which would be considered sensitive to the community.

d) *Were biowaste and radioactive waste disposed of in accordance with local laws?*

All biowaste was disposed in accordance with local laws. There was no radioactive waste generated.

e) *Were any structures built that would have impacted members of the community or the environment (such as handwashing facilities in a public space)? If so, how did you ensure that you had appropriate community buy-in?*

The study refurbished some sections of the public hospitals we were working in. This was achieved after a series of meetings with hospital management personnel and the district health office. They presented a wish list and together with the study team selected what could be achievable with the available resource allocation. The study also provided handwash facilities to public spaces during the covid era. Further, during community awareness campaigns the study supported local sports tournaments as per request from the local communities.

f) *How might the study have impacted existing health-care resources (such as staff workloads, use of equipment that is typically employed elsewhere, or reallocation of public funds)?*

All Research staff worked hand in hand with government staff providing clinical services to all and not limited to study participants only. In sections where there were critical staff shortages such as the maternity unit, extra research nurses were allocated by the local study team to help reduce hospital staff workload. Local study doctors and clinicians were the first line contact for all sick participants or participants requiring out-patient services thus reducing queues and workload for government health care workers. Throughout the study period, the local team conducted malaria and haemoglobin tests, and ultrasound scans for all first ANC visit attendees.

8. Finally, please provide the title (eg, Dr/Prof, Mr/Mrs/Ms/Mx), name, and email address of an author who can be contacted about this statement. This can be the corresponding author.

**Name:** Prof Kamija S Phiri<br>**Email:** kphiri@kuhes.ac.mw
